# Synergistic inhibition effect of diolefinic dye and silver nanoparticles for carbon steel corrosion in hydrochloric acid solution

**DOI:** 10.1186/s13065-024-01298-w

**Published:** 2024-10-05

**Authors:** Marwa N. El-Nahass, Tarek A. Fayed, Mohammed M. El-Gamil, Abd El-Aziz S. Fouda

**Affiliations:** 1 Department of Chemistry, Faculty of Science, Tanta University, Tanta, 31527 Egypt; 2 Department of Toxic and Narcotic Drug, Forensic Medicine, Mansoura Laboratory, Medico Legal Organization, Ministry of Justice, Mansoura, 35516 Egypt; 3 Department of Chemistry, Faculty of Science, Mansoura University, Mansoura, 35516 Egypt

**Keywords:** Corrosion inhibition, Diolefinic dye, Synergism, CS, HCl, Silver nanoparticles, Theoretical calculations

## Abstract

The current work looks at the inhibitory effects of a diolefinic dye, namely 1,4-bis((E)-2-(3-methyl-2,3-dihydrobenzo[d]thiazol-2-yl) vinyl) benzene iodide salt, in relation to CS corrosion mitigation in hydrochloric acid (HCl) environment. This study uses a variety of experimental methodologies, including weight loss (WL) analysis, electrochemical tests, and theoretical considerations. The synergistic effect of diolefinic dye and AgNPs on the corrosion inhibition of CS in 1 M HCl was investigated. The inhibition efficiency (IE) displays a notable enhancement as the concentration of the dye is elevated and as the temperature raises the IE increases. The diolefinic dye exhibited % IE of 83% even at low concentration (1 × 10^–4^ M) whereas 90% in the presence of (2.26 × 10^–10^) AgNPs. Tafel graphs demonstrate that the dye follows a mixed type inhibitor. The adsorption of the dye on CS surface follows Langmuir model. Moreover, the influence of temperature and the activation parameters disclose that diolefinic dye is chemisorbed on the CS surface. The synergistic coefficient of the diolefinic dye and AgNPs under various concentration conditions was greater than unity. The surface morphology of CS sheets was confirmed by scanning electron microscopy (SEM) and energy dispersive X-ray analysis (EDX). Density Functional Theory (DFT) calculations provide theoretical support for the inhibitory effects of the examined dye. Notably, there is a high agreement between the findings of practical studies and theoretical expectations.

**Supplementary Information:**

The online version contains supplementary material available at 10.1186/s13065-024-01298-w.

## Introduction

The widespread use of acid in industrial procedures like pickling, de-scaling, acidizing oil wells, etc., would greatly accelerate the rate at which metals corrode. So, organic inhibitors [1–7] are utilized to protect the metals from acid corrosion. Heteroatom-containing substances have demonstrated good inhibitory effectiveness. In fact, a number of heterocyclic compounds have exhibited remarkable anti-corrosion attributes when exposed to acidic mediums [8–12]. This favorable impact is attributed to the adsorption of these organic moieties onto metal surfaces, which reduces corrosion rates in acidic environments. The adsorption process is a key to these compounds' corrosion inhibition. The efficacy of these compounds as inhibitors stems from their diverse atomic composition, which includes components such as sulphur, phosphorus, nitrogen, oxygen, and double bonds. Diolefinic compounds with heterocyclic subjects, represented by the general formula Ar–CH=CH–Ar–CH=CH–Ar, have found extensive utility in a variety of domains, including laser dyes [13] UV stabilization of polymers [14] electrochromic displays [15] optical imaging devices [16] and electroluminescent devices [17]. Despite these benefits, their potential as corrosion inhibitors has remained untapped and fraught with difficulties.

**Fig. 1:**
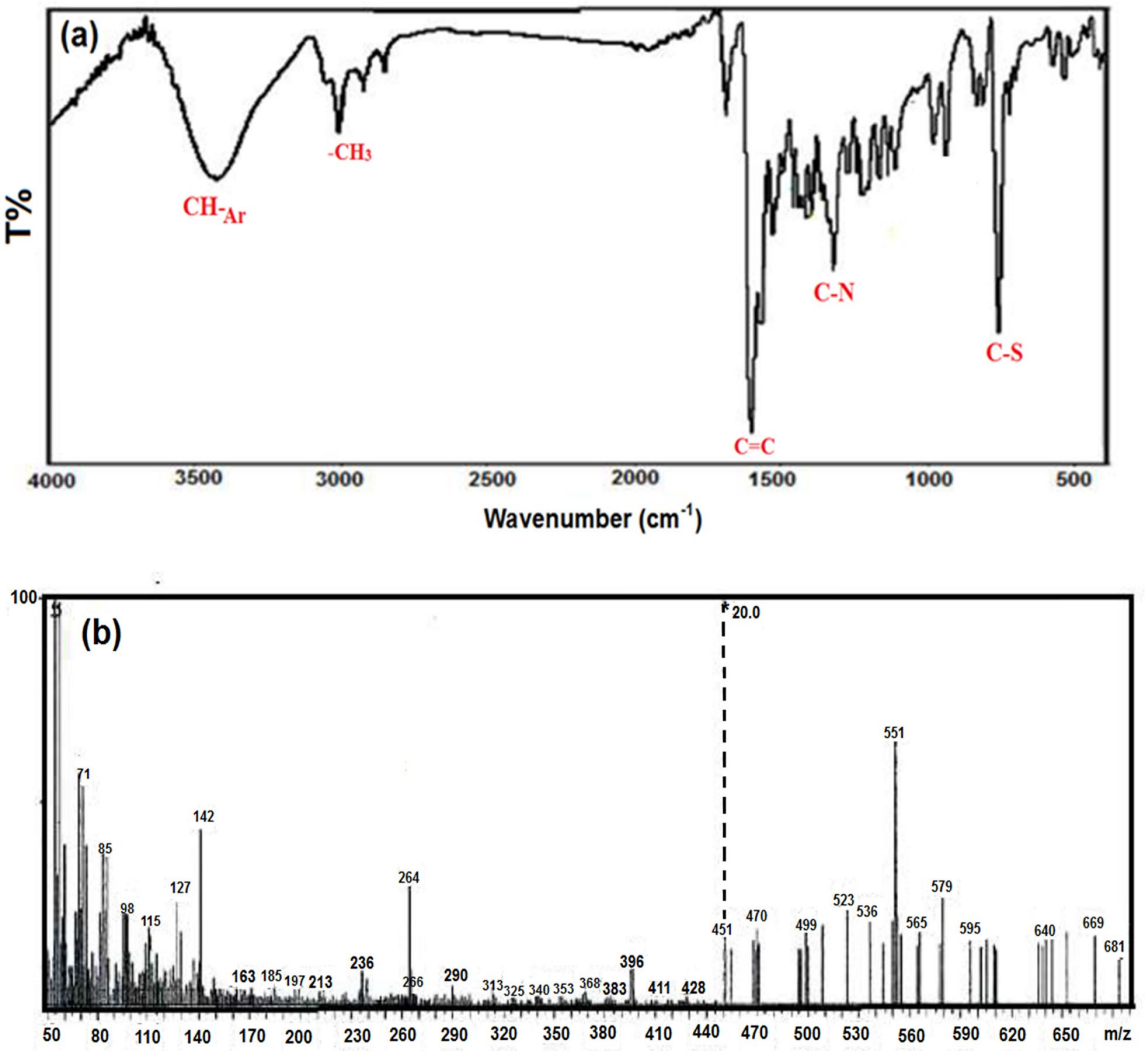
**a** FTIR and **b** Mass spectra of the investigated diolefinic dye, 1,4-bis((E)-2-(3-methyl-2,3-dihydrobenzo[d]thiazol-2-yl) vinyl) benzene, iodide salt

Also, diolefinic dyes are distinguished by their low toxicity often derived from natural or less harmful chemicals, which reduces risks to both humans and wildlife. Biocompatible and conducive to biological applications without disrupting natural processes, they feature a stable chemical structure that minimizes carcinogenic risks compared to traditional synthetic dyes. Environmentally, diolefinic dyes biodegrade more readily, thereby reducing long-term environmental impact and eco-toxicity, particularly benefiting aquatic life and ecosystems [18]. In contrast to azo, anthraquinone, and basic dyes, diolefinic dyes do not release carcinogenic amines, are less toxic, and offer safer alternatives for industrial and biological applications, underscoring their overall environmental and health advantages. Moreover, as corrosion inhibitors for carbon steel, diolefinic dyes provide robust protection by forming a strong, durable barrier through effective adsorption onto metal surfaces. Their stable structure ensures prolonged efficacy at lower concentrations, enhancing cost-effectiveness and environmental compatibility across various environmental conditions, including acidic, neutral, and alkaline environments [19]. The synergetic effect of silver nanoparticles on the corrosion efficiency of organic dyes is notable due to several factors. AgNPs (Silver nanoparticles, generally smaller than 100 nm and contain 20–15,000 silver atoms, have distinct physical, chemical and biological properties compared to their bulk parent materials) are recognized for their antimicrobial properties and capacity to improve the corrosion resistance of materials. When combined with dyes, renowned for their effective corrosion inhibition on CS, they can potentially elevate overall corrosion inhibition effectiveness. This synergy arises from enhanced surface coverage provided by AgNPs, which shields the metal substrate from corrosive agents. Additionally, their antimicrobial action reduces microbial activity on the metal surface, indirectly lowering corrosion rates [20]. Electrochemically, AgNPs may modify the metal surface's behavior, promoting passivation and reducing corrosion rates. Moreover, this combination can enhance the stability of the protective film formed on the metal surface, thereby extending the inhibition effect [21]. Consequently, integrating silver nanoparticles with diolefinic dyes represents a promising avenue for advancing corrosion inhibition strategies on carbon steel. As a result, this study seeks to offer a diolefinic dye, namely; 1,4-bis((E)-2-(3-methyl-2,3-dihydrobenzo[d]thiazol-2-yl) vinyl) benzene iodide salt (Fig. 1), which is intended to inhibit CS corrosion in an HCl environment. Several organic dyes have been used for this purpose [22–30]. The following Table presents a comparative analysis of the inhibition efficiency of the synthesized diolefinic dye in comparison with various related compounds reported in previous studies:

**Table:** 

No	Inhibitor	Corrosive medium	Technique	Optimum Concentration	Efficiency %	Refs.
1	(Z)-4-((2-bromobenzylidene) amino) -5-methyl-2-4-dihydro-3H-1,2,4-triazole-3-thione	1 M HCl	PDP	10^–3^ M	83.66	[22]
	(Z)-4-((3-bromobenzylidene) amino)-5-methyl-2-4-dihydro-3H-1,2,4 -triazole-3-thione				82.84	
2	(E)-2-((2,5-dichlorophenyl)diazenyl)naphthalen-1-ol	0.5 M HCl	PDP	0.075 g	72.2	[23]
3	2-(2-hydroxybenzylideneamino)phenol	1 M HCl	EIS	5 mM	65.0	[24]
	2-(5-chloro-2-hydroxybenzylideneamino)phenol (L2				85.0	
	2-(2-hydroxy-5-nitrobenzylideneamino)phenol				88.0	
4	(E)-4-((2-(2,4-dinitrophenyl)hydrazono)-methyl)pyridine	1 M HCl	PDP	1 mM	80.0	[25]
	(E)-4-(2-(pyridin-4-ylmethylene)hydrazinyl)benzonitrile				78.0	
	(E)-4-((2-(2,4-dinitrophenyl) hydrazono)methyl)phenol				89.0	
5	2-((2-Hydroxyethylimino)methyl)-6-methoxyphenol	1 M HCl	PDP	5 mM	91.3	[26]
			EIS		94.4	
6	2-ercaptothiazoline (2MT)	1 M HCl	PDP	10^−2^ M	93.1	[27]
			EIS		91.1	
7	5-((E)-4-henylbuta-1,3-dienylidene amino)-1,3,4-thiadiazole-2-thiol	1 M HCl	PDP	1 mM	98.2	[28]
			EIS		97.2	
8	Poly(1-phenylethene)	1.0 M HCl	PDP	300 ppm	88.5	[29]
			EIS		89.5	
9	S-Thiazine	1.0 M HCl	PDP	10^–3^ M	91.5	[30]
			EIS		90.9	
10	1,4-bis((E)-2-(3-methyl-2,3-dihydrobenzo[d]thiazol-2-yl) vinyl) benzene iodide salt	1 M HCl	PDP	10^−4^ M	83.0	This study
			EIS		82.0	
	Synergism AgNPs		EIS		90.0	

This study examined the inhibitory impact on CS corrosion in a 1 M HCl solution at various temperatures (298–318 K). In this approach, a variety of chemical and electrochemical procedures were applied. Furthermore, the possible synergistic effect of adding silver nanoparticles (AgNPs) was investigated. To identify the type of protective layer that formed on the CS substrate, the surface morphology was investigated using AFM, EDX, and SEM techniques. Molecular dynamics (MD) simulations were used to get fundamental knowledge regarding the dye's surface adsorption and interactions.

## Experimental

### Preparation of diolefinic dye and AgNPs

#### Synthesis of the studied diolefinic dye

**Scheme 1.**
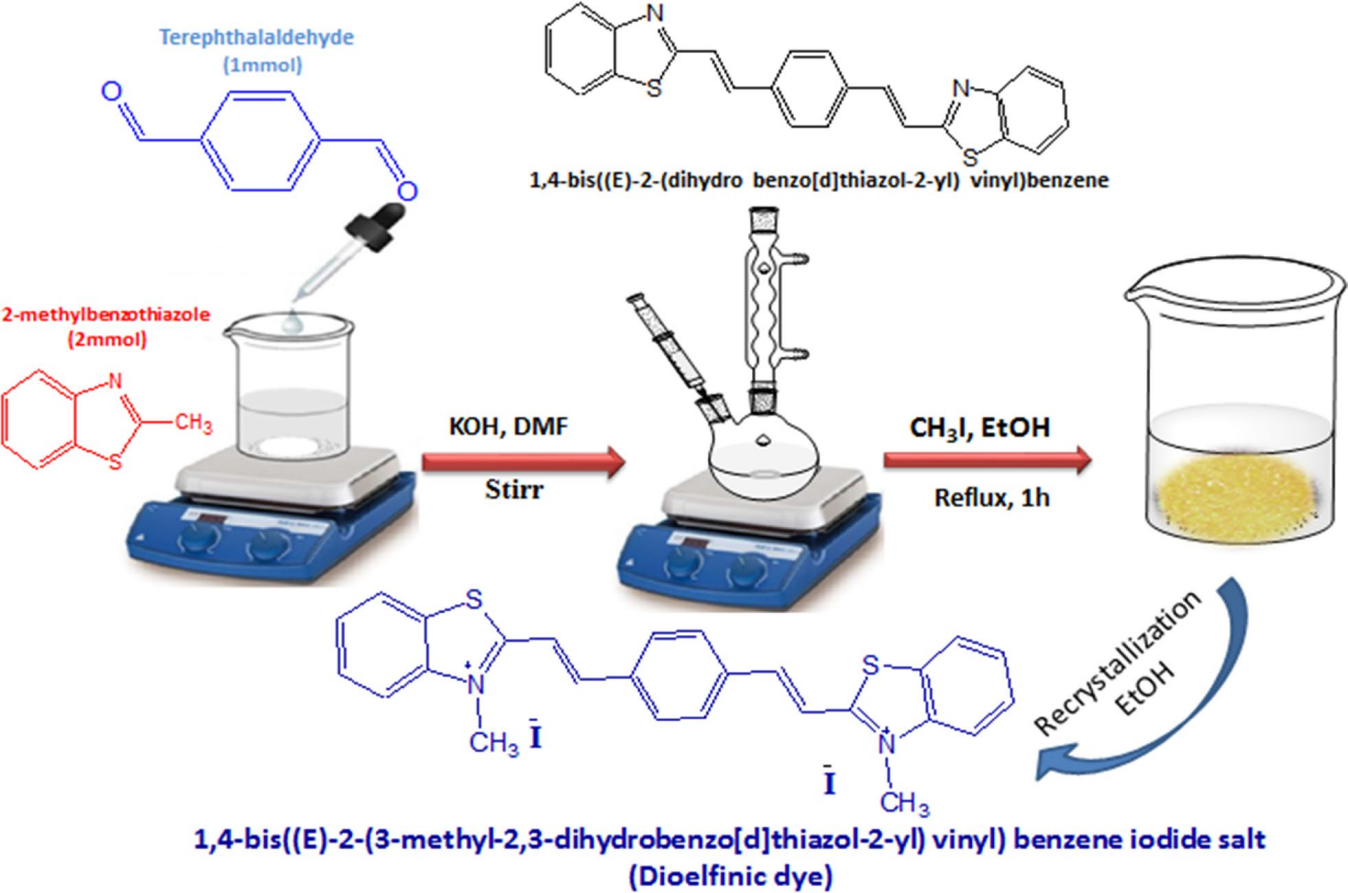
Synthesis of the studied diolefinic dye 1,4-bis((E)-2-(3-methyl-2,3-dihydrobenzo[d]thiazol-2-yl) vinyl) benzene, iodide salt

The investigated dye, namely 1,4-bis((E)-2-(3-methyl-2,3-dihydrobenzo[d]thiazol-2-yl) vinyl) benzene, and its corresponding iodide salt were synthesized following the established methodology for the preparation of this class of diolefinic compounds as outlined previously [31]. Firstly, 1,4-bis((E)-2-(dihydro benzo[d]thiazol-2-yl) vinyl) benzene was synthesized by the condensation of 2-methyl benzothiazole (2 mmol.), with terephthalaldehyde in DMF in the presence of KOH. Heating of the obtained product, for 1 h under reflux, with equimolar quantity of methyl iodide in ethanol yields the investigated diolefinic dye, 1,4-bis((E)-2-(3-methyl-2,3-dihydrobenzo[d]thiazol-2-yl) vinyl) benzene, iodide salt. It underwent two rounds of recrystallization using ethanol as the solvent. Subsequently, the material underwent vacuum sublimation under light-protected conditions, Scheme 1. Yield (82%); yellow crystals; M.P: 125 °C; IR: ν/cm^−1^: 3423 (CH-_Ar_), 3007 (–CH_3 Aliph_), 982 (CH=CH), 1571 (C=C), 1322 (C–N), 762 (C-S). MS (EI) *m*/*z*: calcd for C_26_H_22_I_2_N_2_S_2_ [M]^+^, 680.93; found, 681 (20.0%).

#### Synthesis of AgNPs

**Scheme 2.**
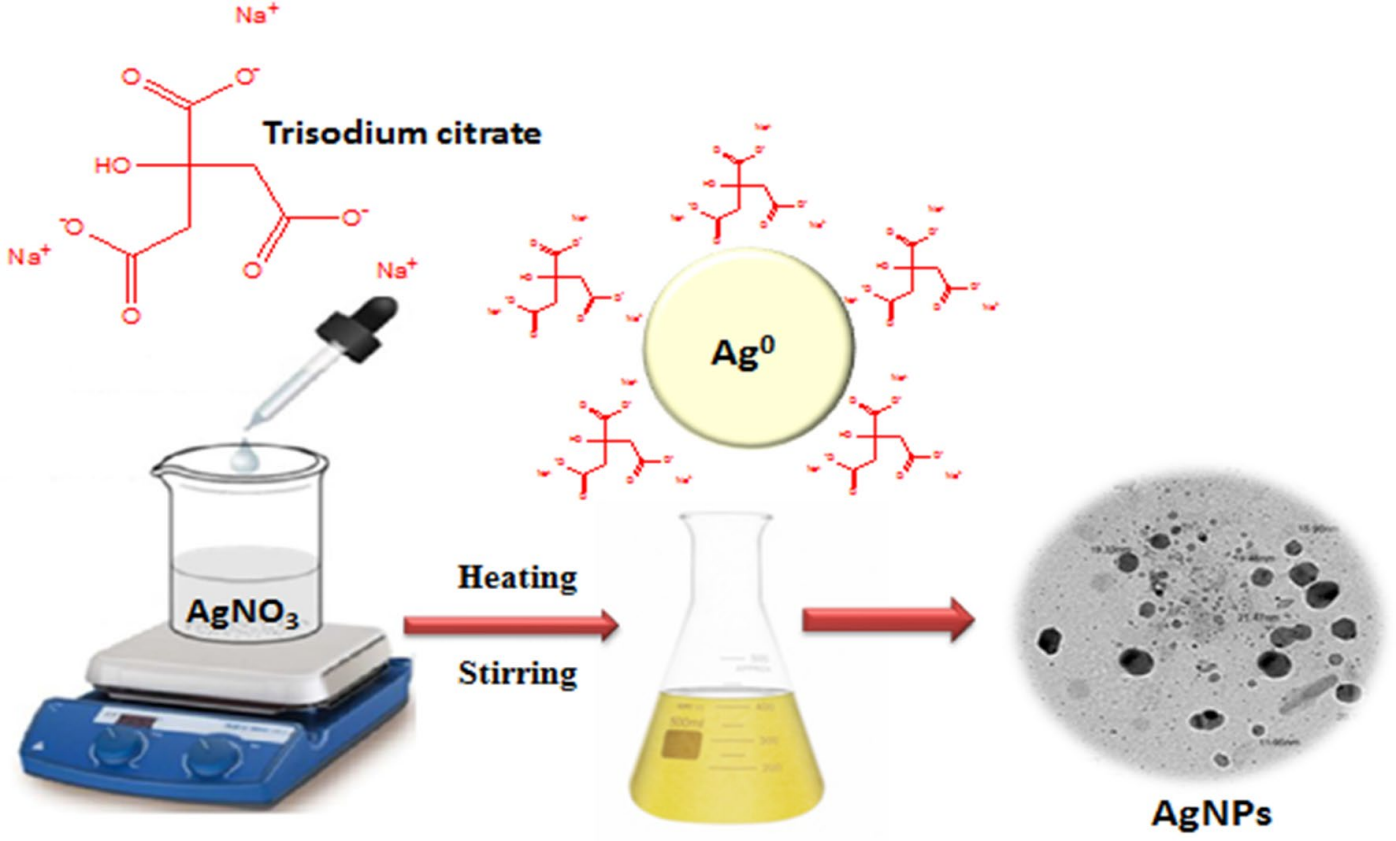
Synthesis of silver nanoparticles (AgNPs)

The following approach was used to create colloidal silver nanoparticles (AgNPs) in distilled water: To begin, 50 mL of 1 × 10^−3^ M AgNO_3_ was heated to boiling. Concurrently, 4 mL of a 1% trisodium citrate solution was carefully added dropwise to the AgNO_3_ solution under continuous stirring and heating until the solution's color changed to yellow, demonstrating the creation of Ag nanoparticles, Scheme 2**.** The nanoparticles formed because of citrate's reduction of Ag^+^ to Ag^0^, which was confirmed by the observed change in color. Following that, the Ag nanoparticle solution could gradually cool to ambient temperature before being conserved at 4 °C. The resultant Ag nanoparticles, synthesized through this procedure, were subsequently subjected to comprehensive characterization using transmission electron microscopy. The concentration of these synthesized nanoparticles was ascertained employing a previously validated method [32] yielding a value of 1.64 × 10^–9^ M.

### Materials

In this study, the chemical reagents were procured from commercial suppliers and used without further purification. 2-Methylbenzothiazole, terphthaldehyde, methyl iodide and potassium hydroxide were sourced from Sigma-Aldrich Chemical Co. (St. Louis, MO, USA). The chemical composition of carbon steel operated as working electrode in this study is (weight percent): C 0.2, Mn 0.035, Si 0.300, S 0.021, P 0.003, and Fe made up the remaining portion were used for all measurements employed to monitor its corrosion. The metal sheets were mechanically cut into square coupons of dimensions 20 mm × 20 mm × 1 mm for gravimetric method and 10 mm × 10 mm × 1 mm for electrochemical analysis. For electrochemical tests the CS sheet was welded with Cu-wire for electrical connection and inserted into Teflon tube and fixed with an adhesive [33]. Exposed areas were scraped utilizing various grades of emery papers with different grit range, followed by degreasing with acetone, rinsed by double distilled water and finally dried.

### Preparation of solutions

In this study, corrosive solutions were prepared by diluting the analytical reagent (AR) grade HCl at a concentration of 37% by double distilled water. The dye's stock solution (10^–3^ M) was prepared using ethanol. The corrosion investigations were carried out utilizing one molar HCl solution with and without different dye concentrations. Dye spanning from 1 × 10^−6^ to 1 × 10^–4^ M, as well as/or AgNPs. Every experiment employed brand-new solutions, and they were all carried out at 298 K in an air environment without stirring.

### WL methods

Prior to commencing each examination, the specimens underwent a thorough cleansing process using double distilled water, followed by successive abrasion using emery papers with varying degrees of coarseness. Subsequently, the samples were weighed and then subjected to immersion in 100 mL of one molar HCl solution having altered doses of the diolefinic dye under test. These measurements were conducted across a range of temperatures (298 to 318 K), while maintaining constant immersion duration of 3 h within a temperature-regulated water bath. After an interval of 30 min, the CS samples were detached from the solution, rinsed with double distilled water, and allowed to gradually air dry before undergoing reweighing. The parameters of WL such as % IE, Ɵ, and CR pertaining to the investigated dye were determined through the following mathematical expressions [34].

1 $$\upsilon \, = \, \Delta {\text{W}}/{\text{Sxt}}$$

2 $$\% {\text{ IE }} = \, \left( {\upsilon_{0} {-} \, \upsilon } \right) \, /\upsilon_{0}$$

where ΔW denotes the average weight loss, S represents the sample surface area (cm^2^), t stands for the time of immersion (min), ʋ_o_ and ʋ designate the values of corrosion rate (mg cm^−2^ min^−1^) uninhibited and inhibited media, respectively.

### Electrochemical measurements

During this investigation, a CS350 Electrochemical Workstation was used with a classical three-electrode system: CS as working electrode (~ 1 cm^2^), 3 mol/L Ag/AgCl as a reference electrode and large surface area sheet of Pt as an auxiliary electrode. The CS was maintained immersed in the corrosive solution until the open-circuit potential (OCP) was stabilized (30 min) before carrying the electrochemical tests. The PDP curves were plotted by a scan rate of 1 mV/s and the range of OCP ± 250 mV. The EIS was recorded at various frequencies between 10^5^ Hz and 10^–2^ Hz in the OCP with amplitude of 10 mV. All the tests were approved in stagnant aerated solution and at least three times for reproducibility.

### SEM and EDX analysis

The surface analyses were executed through SEM and EDX. CS sheets were dipped in one molar HCl solutions containing both low and high doses of the studied dye for a period of 24 h. In cases involving a combination of the dye and silver nanoparticles (Ag NPs) solution, the surfaces of the CS samples were scrutinized using a scanning electron microscope, specifically the JEOL JSM 639 model.

### Computational investigation

DFT calculations were performed using the Materials Studio software (version 20.1, developed by BIOVIA Inc.) in conjunction with the DMol3 module [34, 35]. The DMol3 software employs numerical functions that are atom-centered, providing a more comprehensive representation compared to traditional Gaussian functions. Geometrical optimizations were carried out utilizing the B3LYP hybrid functional, which combines the Becke-3-exchange and Lee–Yang–Parr correlation, along with the DNP basis set for double numerical with polarization. In order to account for solvent effects, specifically employing water with a dielectric constant of 78.54, the conductor-like screening model (COSMO) [36] was employed to enhance the reliability of the obtained results. The investigation of the adsorption process of an inhibitor onto a surface comprised of iron was conducted using Monte Carlo simulations with the utilization of the adsorption locator module developed by Materials Studio software (version 20.1, developed by BIOVIA Inc.) [37]. The selection of the Fe (110) surface as the substrate for replicating the adsorption process was driven by its favorable structural characteristics. Among the available Fe surfaces, including Fe (110), Fe (100), and Fe (111), Fe (110) was chosen due to its densely packed structure and superior stability [38, 39]. To create this surface, a body-centered cubic (bcc) Fe crystal was cleaved and expanded into an (8 × 8) supercell, followed by the establishment of a vacuum slab with a thickness of 25 Å above the Fe (110) plane. The study involved simulating the interaction between the Fe (110) surface and specific inhibitor monomer molecules, designated as 1–8 #inhibitor monomers, in a cubic simulation box with dimensions of 19.809 Å × 19.809 Å × 31.065 Å. This simulation aimed to investigate the impact of varying inhibitor concentrations. In order to achieve an equilibrium configuration for the inhibitor/Fe (110) system, we employed simulated annealing to facilitate the adsorption of optimized inhibitor molecules onto the refined Fe (110) surface. Subsequently, we determined the adsorption energy for the most stable configuration of the inhibitor/Fe (110) system. To account for the influence of solvent effects; we adopted the COMPASS-III (Condensed-phase Optimized Molecular Potentials for Atomistic Simulation Studies) methodology. Within this framework, we conducted structural optimization of inhibitors, incorporating a solvent environment consisting of 380 H_2_O, 5H_3_O^+^, and 5HCl molecules in the adsorption system, with or without the presence of 4Ag molecules. This innovative application represents a notable advancement in forcefield methodology [40]. It is noteworthy that COMPASS-III stands as an ab-initio forcefield, capable of providing predictions pertaining to chemical attributes such as structural, conformational, and vibrational properties, as well as condensed-phase properties encompassing the equation of state and cohesive energies, for a wide range of chemical systems.

## Results and discussion

### Characterization of the investigated diolefinic dye and AgNPs

**Fig. 2:**
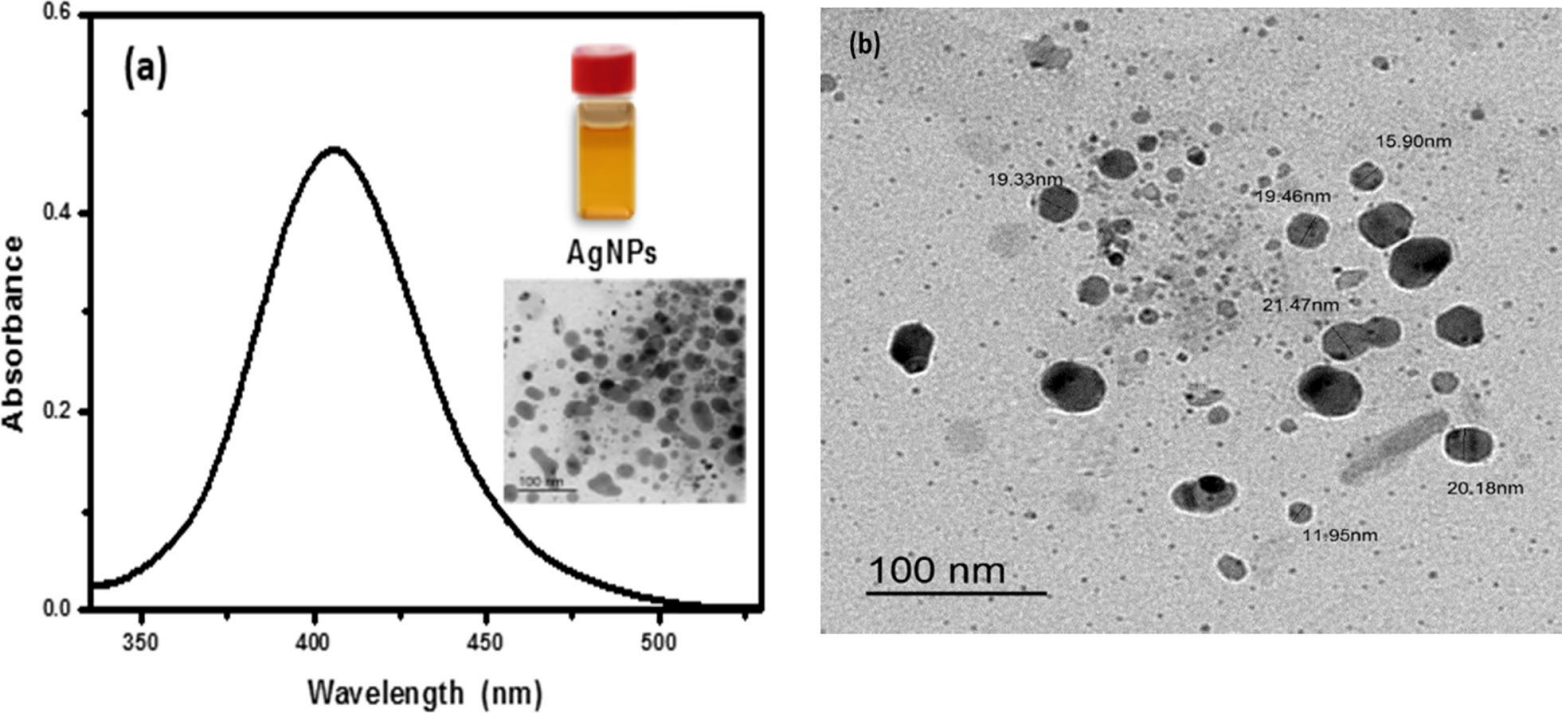
**a** Absorption spectrum and **b** Transmission electron micrographs of synthesized Ag NPs of average size, 18.05 nm diameter

**Fig. 3:**
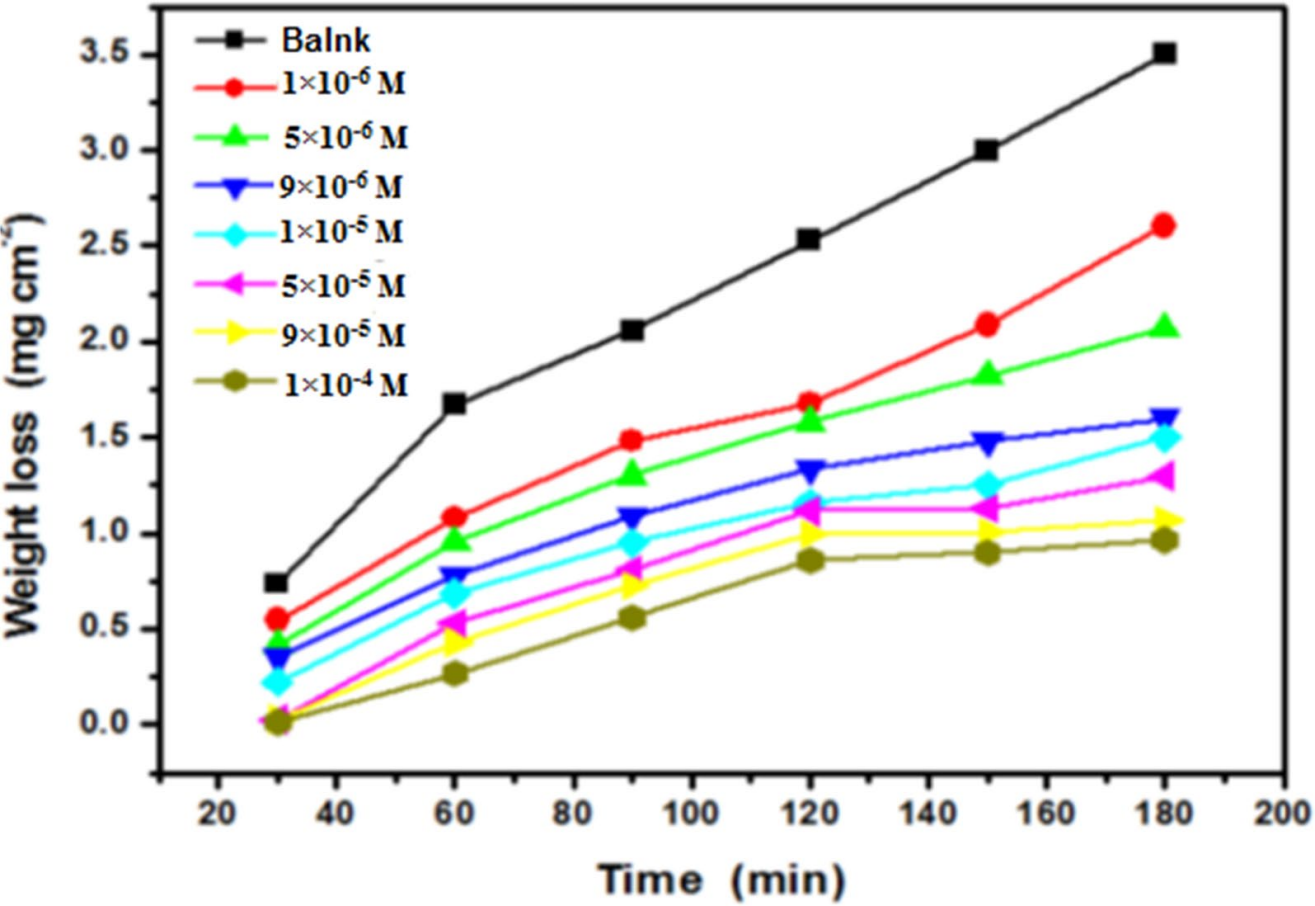
Weight loss-time curves for the corrosion of carbon steel in 1 M HCl in the absence and presence of different concentrations of diolefinic dye (1 × 10^–6^–1 × 10^–4^ M) at 298 K

The diolefinic dye’s structural characterization was “achieved through the utilization of mass spectrometry and Fourier-transform infrared spectroscopy (FT-IR). The FT-IR analysis revealed distinct spectral features, specifically vibrational peaks at wavenumbers of 762, 1322, and 1571 cm^−1^, indicating the presence of C–S, C–N, and ethylene double bonds, respectively, as depicted in Fig. 2a. Additionally, an observable peak at 982 cm^−1^ was attributed to the out-of-plane bending vibration of the CH=CH moiety. Furthermore, a broad band centered at 3423 cm^−1^, corresponding to the stretching vibrations of aliphatic C–H groups, was evident in the spectra. The mass spectrometric analysis of the diolefinic dye exhibited a molecular ion peak at the anticipated position of m/z = 682, as illustrated in Fig. 2b. This molecular ion peak corroborated the proposed molecular weight of the dye. The absorption spectra of silver nanoparticles (AgNPs) within the wavelength range of 300–580 nm was illustrated in Fig. 3a. A distinct and sharp peak at 404 nm was ascribed to the surface Plasmon resonance band, which aligns with the characteristic of AgNPs exhibiting a fully or nearly spherical shape. Conversely, Fig. 3b showcased typical transmission electron microscope (TEM) images of the AgNPs, revealing their approximately spherical shape along with an irregular distribution pattern. The average particle size was determined to be 18.05 nm.

### Chemical method (gravimetric tests, WL)

**Fig. 4:**
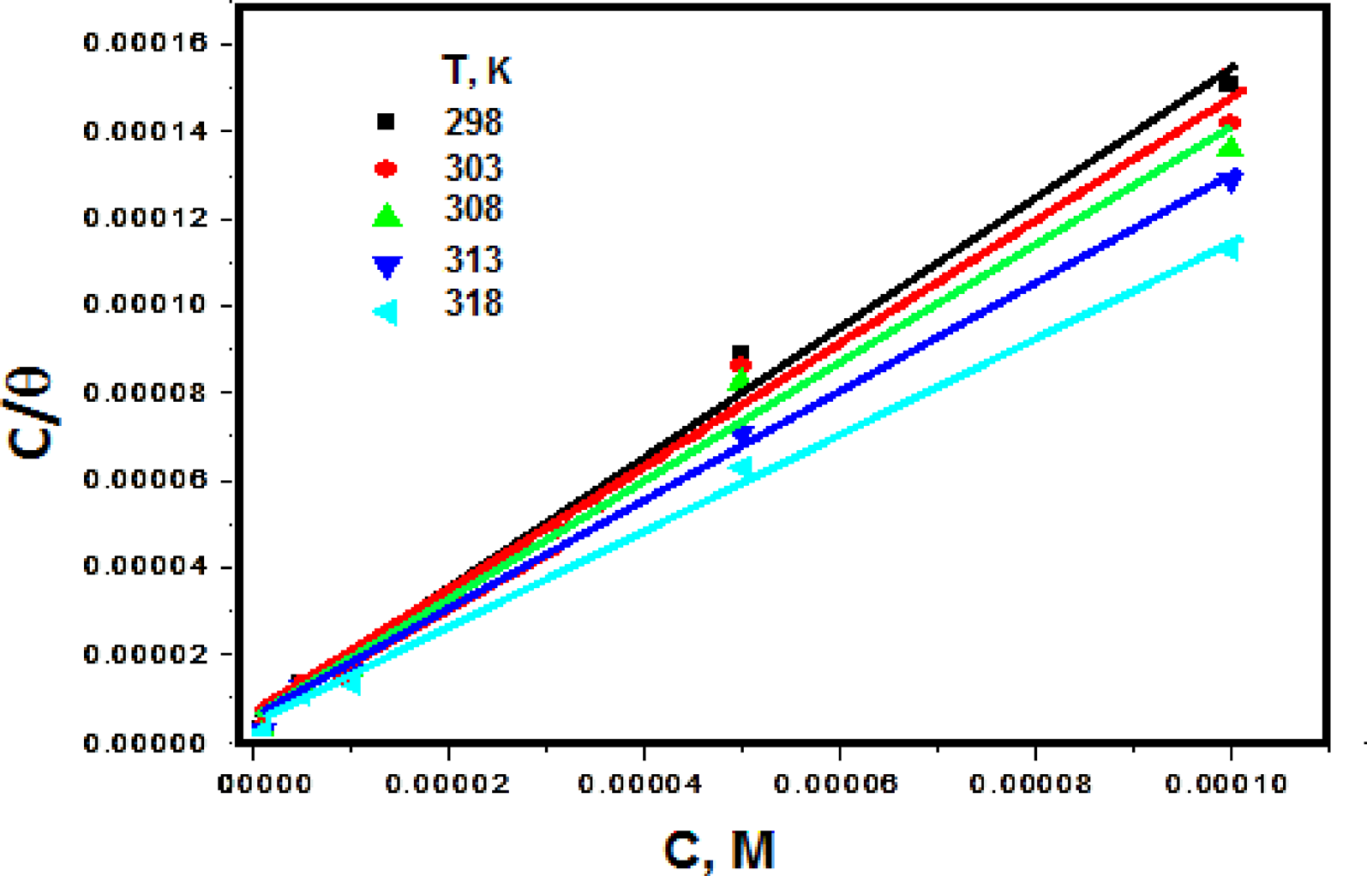
Langmuir adsorption isotherm of diolefinic dye on carbon steel surface in 1 M HCl at different temperatures (298–318 K)

Figure 4 illustrates the WL over time for CS in one molar HCl, comparing situations with varying concentrations of the diolefinic dye at 298 K. These WL curves exhibit an initial rapid increase. The presence of the additive results in lower weight loss compared to the free acid situation, indicating that both the inhibitor type and concentration influence CS weight loss. Table 1 presents data indicating a positive correlation between the percentage of inhibitor efficiency (IE) and higher concentrations within a corrosive environment. This observation suggests that increased bulk concentration and surface coverage of the additive lead to a deceleration of CS dissolution. The investigation also encompassed an examination of the influence of temperature on the corrosion rate of CS in the presence of various concentrations of diolefinic dye within the 298 to 318 K. Table 2 demonstrates that as temperature escalates, the corrosion rate diminishes, while the percentage of inhibitor efficiency (IE) of the dye rises. The results can be explained by an improvement in the adsorption of dye particles onto the CS surface. The adsorption behavior of the dye on CS surfaces is primarily governed by physical adsorption processes.

**Table 1:** The inhibition efficiency (IE) and surface coverage (θ) of the examined diolefinic dye for the corrosion of carbon steel in 1 M HCl. from weight-loss measurements at different concentrations

[Inhibitor] M	θ	% IE
1 × 10^–6^	0.336	33.6
5 × 10^–6^	0.374	37.4
9 × 10^–6^	0.469	46.9
1 × 10^–5^	0.541	54.1
5 × 10^–5^	0.558	55.8
9 × 10^–5^	0.605	60.5
1 × 10^–4^	0.659	65.9

**Table 2:** Values of IE of diolefinic dye for carbon steel corrosion in 1 M HCl derived from weight-loss experiments at different concentrations and temperatures

[Diolefinic dye] M	298 K	303 K	308 K	313 K	318 K
1 × 10^–6^	33.6	34.61	37.95	39.10	41.74
5 × 10^–6^	37.4	38.18	40.44	0.40	45.42
9 × 10^–6^	46.9	48.67	52.38	56.79	64.47
1 × 10^–5^	54.1	54.91	58.95	61.50	73.73
5 × 10^–5^	55.8	57.94	60.56	70.51	79.67
9 × 10^–5^	60.5	61.41	62.90	73.18	83.31
1 × 10^–4^	65.9	70.10	73.53	77.29	88.08

### Adsorption isotherm

**Fig. 5:**
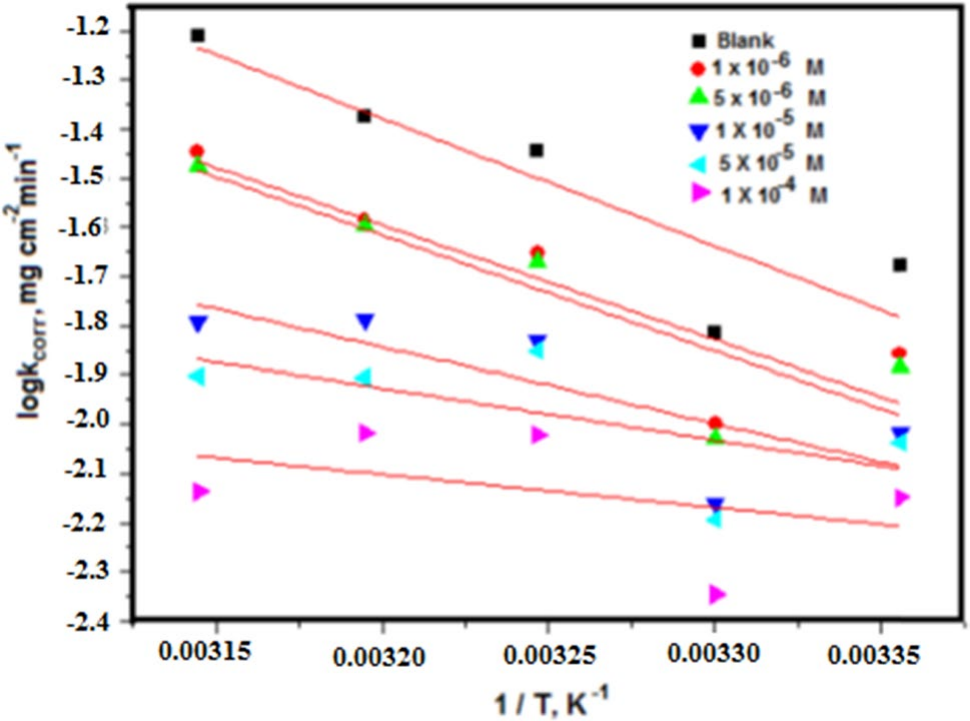
Plots of (log k_corr_) against (1/T) in the case of diolefinic dye in 1 M HCl

The adsorption isotherm, an invaluable quantitative representation of adsorption phenomena [41], was originally developed for characterizing the metal-inhibitor-environment system. Consequently, the obtained results were examined through the utilization of various adsorption isotherm models, including the Langmuir, Temkin, Frumkin, and Freundlich isotherms”. It is evident that the correlation between the Langmuir isotherm and the data is almost equivalent to unity (R^2^ > 0.99) (Fig. 5), as indicated by the following Eq. [42].

3 $${\text{C }}/ \, \theta \, = { 1}/{\text{ K}}_{{{\text{ads}}}} + {\text{ C}}$$

where (K_ads_) donates the adsorption equilibrium constant, (θ) signifies the surface coverage and C refer to dye’s concentration. The graphical representation of Fig. 5 displayed linear plots with a nearly unity slope, implying that the dye adhered to the metal surface in accordance with the Langmuir adsorption isotherm. According to this particular isotherm, there exist no intermolecular interactions among the adsorbed species, each occupying a separate site [43]. The high K_ads_ values (K_ads_ = 19–27 × 10^4^ mol/L) **(**Table 3**)** suggest that the dye has strongly adsorbed to the surface of the CS [44, 45]. “To further understand the dye adsorption mechanism, the standard adsorption free energy (∆G^o^_ads_) associated with (K_ads_) was computed using the following equation [46]”:

4 $${\text{K}}_{{{\text{ads}}}} = { 1}/{55}.{\text{5 exp }}( - \Delta {\text{G}}^{0}_{{{\text{ads}}}} /{\text{ RT}})$$

**Table 3:** Thermodynamic parameters for the adsorption of diolefinic dye on carbon steel surface in 1 M HCl at different temperatures

Temperature K	K_ads_ × 10^6^ M^−1^	ΔG_ads_ K.J mol	ΔH_ads_ K.J mol	ΔS_ads_ K.J mol
298	0.19	− 40.1	14.7132	184.1
303	0.17	− 40.5		182.4
308	0.19	− 41.5		182.5
313	0.23	− 42.6		183.3
318	0.27	− 43.7		183.7

The term “mol/L” denotes the molar concentration of water within the solution, specifically at a value of 55.5 in the present context. The negative values of ΔG^o^_ads_ ensure the spontaneity of the adsorption process and stability of the adsorbed film over the steel surface [47]. According to literature, values of ΔG^o^_ads_ near to – 40 kJ/mol. or more − ve imply a charge transfer between the dye molecules and the metallic substrate [48, 49]. On the other hand, values of − 20 kJ/mol. or higher indicate the presence of charged molecules in the interaction with charged metals (physical adsorption). The inhibitor under research (ΔG◦_ads_ >  − 40 kJ/mol) is chemisorbed onto CS, according to the ΔG◦_ads_ data (Table 3).

### Corrosion parameter with kinetic and thermodynamic attributes

The elucidation of activation parameters served to elucidate the underlying process governing the communication between dye molecules and the surface of CS. To quantify the thermodynamic activation parameters associated with the dissolution of CS in a one molar HCl solution within 298 to 318 K, Arrhenius and transition-state Eqs. (5 & 6) were employed. These computations were conducted both in the presence and absence of the utilized dye.

5 $${\text{k}}_{{{\text{corr}}}} = {\text{A exp }}\left( { - {\text{E}}_{{\text{a}}}^{*} /{\text{RT}}} \right)$$

6 $${\text{Log k}}_{{{\text{corr}}}} /{\text{T }} = {\text{ log }}({\text{R}}/{\text{ Nh }} + {\text{DS}}^{*} /{ 2}.{3}0{\text{3R}}) \, + \, ( - {\text{DH}}^{*} /{ 2}.{3}0{\text{3R}}){ 1}/{\text{ T}}$$

**Fig. 6:**
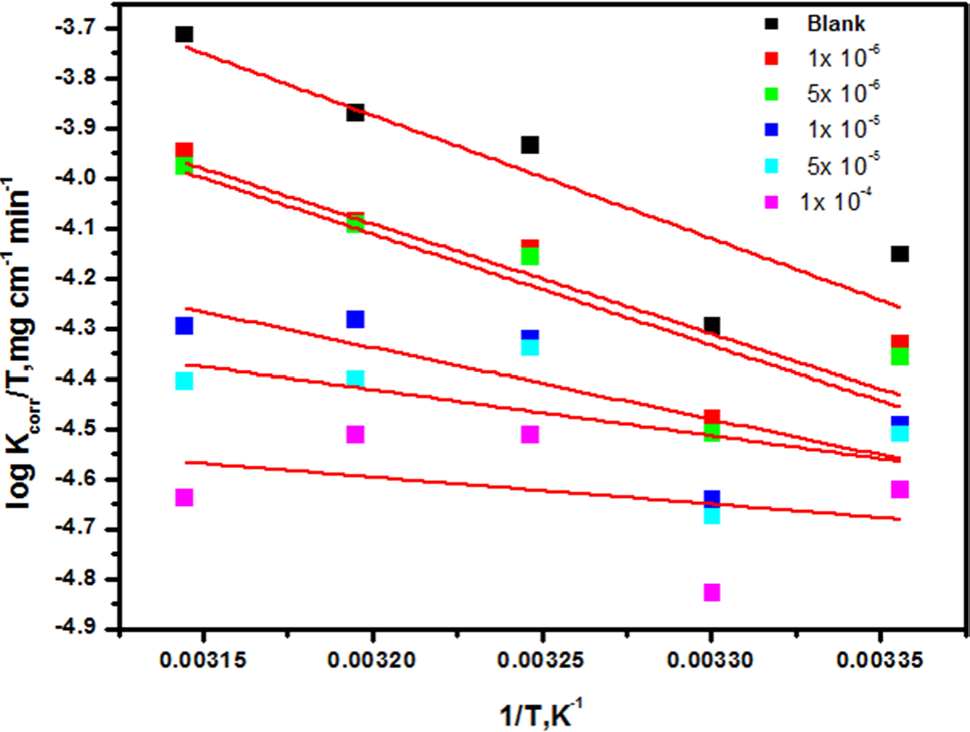
Plots of (log k_corr_/T) against (1/T) in the case of diolefinic dye in 1 M HCl

**Fig. 7:**
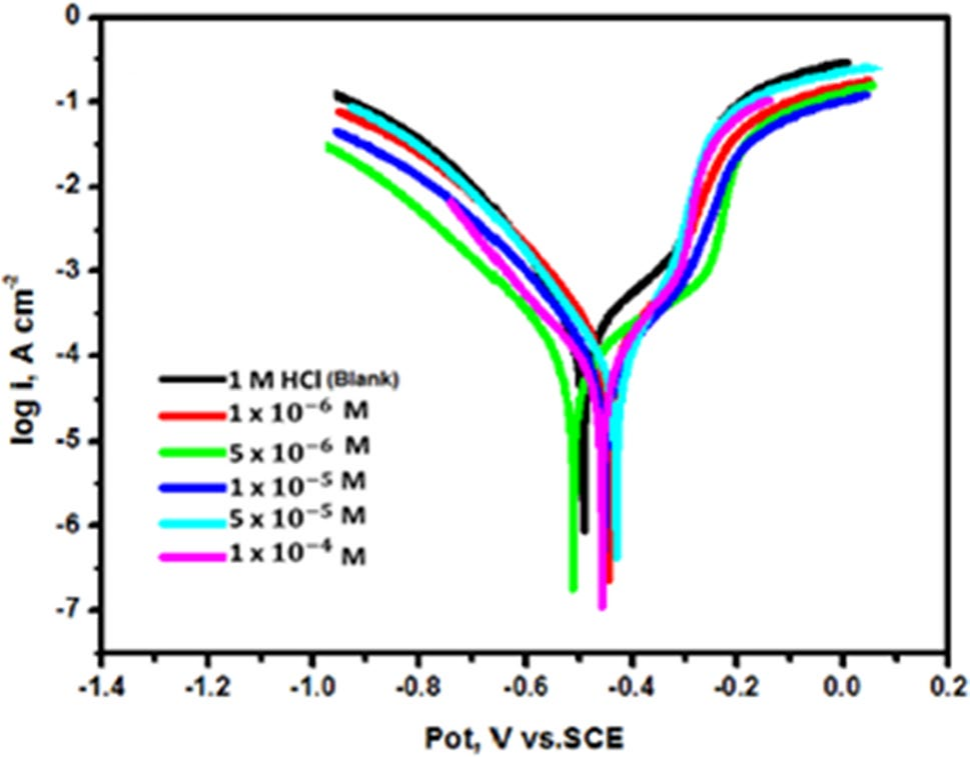
Potentiodynamic polarization curves for corrosion of carbon steel in 1 M HCl in the absence and presence of different concentrations of diolefinic dye at 298 K

E_a_^*^, ∆H^*^, and ∆S^*^ are employed to represent the activation energy, activation enthalpy, and activation entropy, respectively, while R signifies the universal gas constant, N denotes Avogadro's number, and h stands for the Planck constant. In Fig. 6, a linear graphical representation is presented, illustrating the correlation between the natural logarithm of the corrosion rate (log k_corr_) and the reciprocal of the absolute temperature (1/T) for CS immersed in a 1 M HCl solution. Through an examination of the gradient values at various temperatures, it becomes feasible to ascertain the Arrhenius activation energy (E_a_^*^). The application of the Arrhenius-type model facilitated the computation of kinetic parameters associated with the corrosion of CS [50]. The graphical representation of the relationship between the logarithm of (log k_corr_/T) and (1/T) produces linear plots characterized by slopes equivalent to (ΔH^*^/2.303R) and intercepts denoted as [log (R/Nh + ΔS^*^/2.303R)]”, as illustrated in Fig. 7. Table 4 provides comprehensive data on the apparent activation energy, activation enthalpy, and activation entropy of corrosion for CS in a 1 M HCl solution under varying concentrations of diolefinic dye. Notably, the activation energy values (E_a_^*^) for the dye were observed to be lower than those of the blank solution, suggesting that adsorption predominantly occurs at active sites possessing higher energies, while corrosion predominantly occurs at active sites with lower energies. This observation implies that the dye is typically chemisorbed on the surfaces of CS [51]. Additionally, the positive values of ΔH^*^ verify that the process of forming the activated complex is endothermic, but the negative values of ΔS^*^ suggest that the reaction progresses with a higher order of magnitude as reactants are converted into the activated complex [52]^.\^

**Table 4:** Activation parameters for carbon steel corrosion in the absence and presence of various concentrations of diolefinic dye in 1 M HCl

Concentration M	Ea* kJmol^−1^	ΔH* kJ mol^−1^	Δs* J mol^−1^
Blank	49.7	20.4	− 0.12
1 × 10^–6^	44.6	18.2	− 0.14
5 × 10^–6^	45.1	18.4	− 0.14
1 × 10^–5^	29.8	11.8	− 0.19
5 × 10^–5^	20.1	7.6	− 0.22
1 × 10^–4^	12.9	4.4	− 0.25

Asterisk means activation

Ea* is the activation energy

ΔH* is the activation enthalpy

Δs* is activation entropy

### Electrochemical studies of the investigated diolefinic dye and the synergistic Ag nanoparticles

#### Electrochemical PDP analysis

**Fig. 8:**
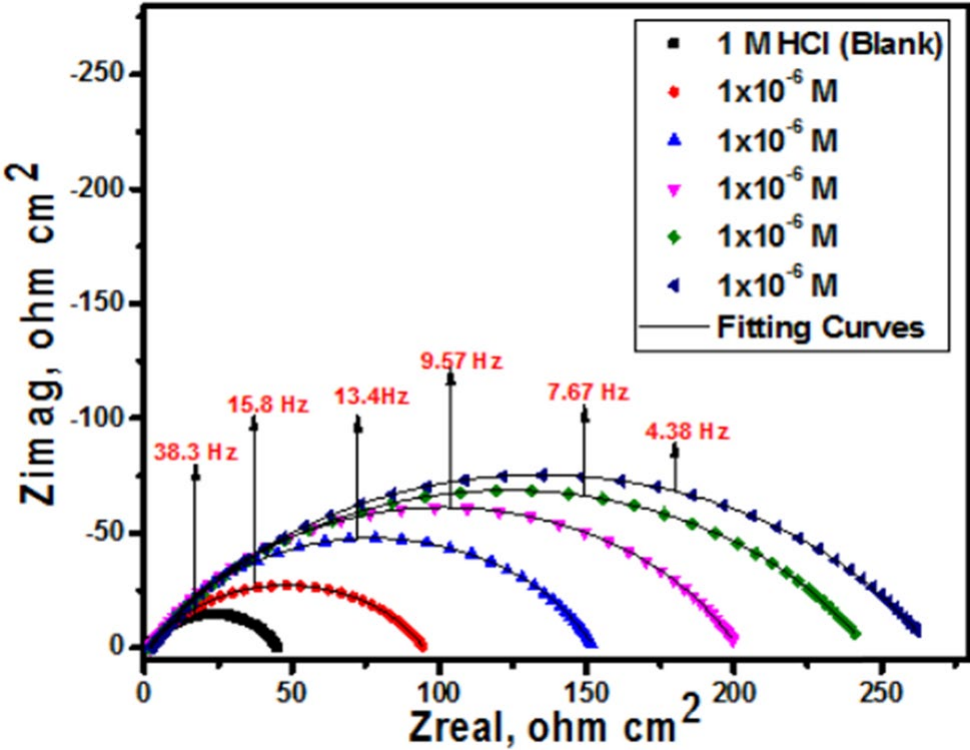
The Nyquist plots for the corrosion of carbon steel in 1 M HCl in the absence and presence of different concentrations of diolefinic dye at 298 K

PDP is a crucial analytical technique in studying corrosion inhibition because it provides valuable information about the corrosion rate and the tendency for pitting or passivation of a material. It also helps quantify the effectiveness of corrosion inhibitors by comparing the polarization curves obtained in the presence and absence of inhibitors. This technique is preferred in corrosion inhibition studies because it provides comprehensive data on inhibitor performance, mechanisms of protection, and real-time corrosion monitoring, which are essential for developing effective corrosion control strategies. Figure 8 depicts the PDP curves of CS immersed in a 1M HCl solution with and without altered concentrations of diolefinic dye. It is evident from the Figure that the addition of the diolefinic dye to the aggressive media reduces both the anodic metal dissolution and cathodic hydrogen evolution processes. As the quantity of diolefinic dye increases, the suppression of these processes becomes more noticeable. These findings suggest that the inhibitor under investigation functions as a mixed type corrosion inhibitor [44]. At each dose, the anodic Tafel slope (β_a_) data show a higher displacement than the cathodic Tafel slope (β_c_) values, confirming the inhibitor's anodic region of Tafel behavior.

Table 5 demonstrates that there is no significant difference in the E_corr_ values and Tafel slopes with and without solutions containing inhibitor. These findings strongly suggest that the investigated dye function as a mixed-type inhibitor [53]. Moreover, the incorporation of the dye leads to noticeable alterations in both cathodic (β_c_) and anodic (β_a_) Tafel constants, causing them to shift towards more negative and more positive values, respectively. This phenomenon indicates an unaltered corrosion reaction mechanism [49]. We performed electrochemical study using Tafel plots to establish essential electrochemical parameters such as corrosion current density (icorr), corrosion potential (E_corr_), Tafel constants (β_a_ and β_c_), (θ), and (IE). To determine the inhibitor's (θ) and (IE), Eq. (7) was used:

7 $$\% {\text{ IE}} = \, \theta \times {1}00 = \left[ {{1} - \left( {{\text{i}}_{{{\text{corr}}}} /{\text{i}}_{{{\text{corr}}}}^{{^{{\text{o}}} }} } \right)} \right] \times {1}00$$

**Table 5:** Corrosion potential (E_corr_), corrosion current density (i_corr_), Tafel slopes (β_c_, β_a_), degree of surface coverage (θ), and inhibition efficiency (IE) of carbon steel in 1 M HCl at 298 K for diolefinic dye

[Diolefinic dye], M	− E_corr_ mV vs. Ag/AgCl	i_corr_ μA cm^−2^	β_a_ mV dec^−1^	− β_c_ mV dec^−1^	k_corr_ mpy	ϴ	% IE
Blank	492	223	190.91	127.9	2.61	–	–
1 × 10^–6^	445	134	138.3	135.51	1.57	0.390	39.0
5 × 10^–6^	512	109	292.13	161.61	1.28	0.510	51.0
1 × 10^–5^	453	81	151.66	136.87	0.95	0.630	63.0
5 × 10^–5^	431	58	80.59	125.57	0.78	0.730	73.0
1 × 10^–4^	458	38	90.02	124.78	0.45	0.820	82.0

The corrosion currents, namely i_corr_ and i^o^_corr_, correspond to the corrosion currents observed with and without of corrosion inhibitors, respectively. By extending the straight portions of the anodic and cathodic curves to the locations where the respective corrosion potentials intersect, the dissolution current densities were computed. The obtained results manifest a noteworthy reduction in the dissolution current density (i_corr_) when inhibitor is introduced.

#### EIS measurements

**Fig. 9:**
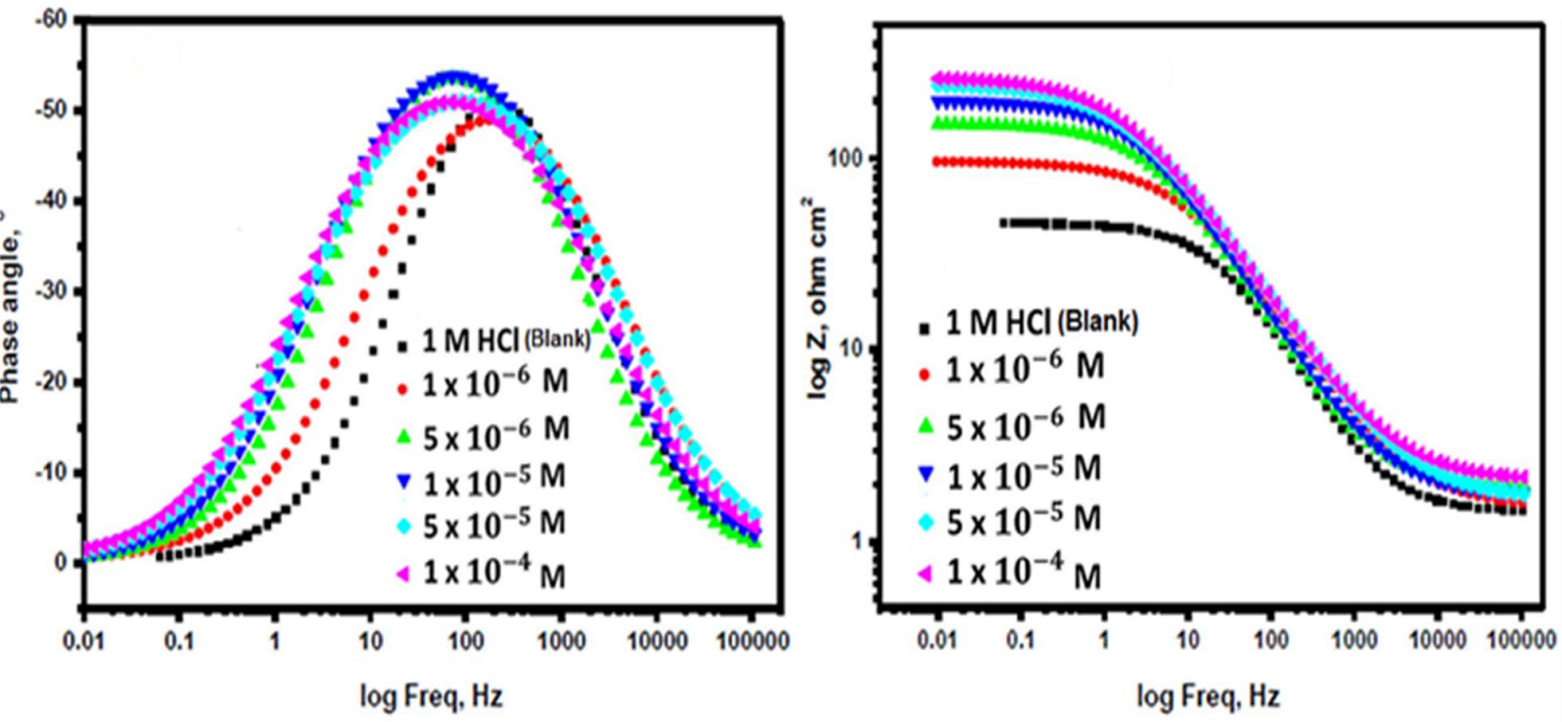
The Bode plots for the corrosion of carbon steel in 1 M HCl in the absence and presence of different concentrations of diolefinic dye at 298 K

**Fig. 10:**
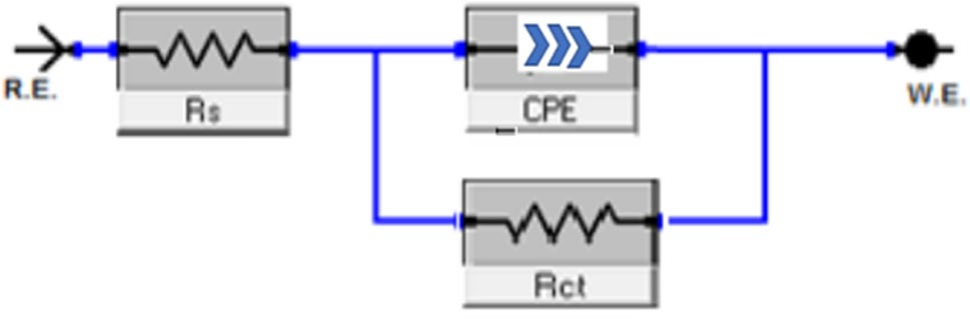
Electrical equivalent circuit model used to fit the results of impedance

EIS stands as a well-established and efficacious methodology in the realm of corrosion investigation [54–58]. It provides information about the electrical properties of the corrosion interface, including charge transfer resistance, double-layer capacitance, and the presence of protective films of inhibitors. This helps in understanding how inhibitors interact with the metal surface and hinder the corrosion process. Utilizing impedance plots facilitates the derivation of critical surface parameters, electrode kinetics, and mechanistic insights. In the context of CS dissolution in one molar HCl at 298 K, the impact of inhibitor concentration is intensely illustrated in Figs. 9 and 10 specifically; Fig. 9 demonstrates a Nyquist plot depicting the behavior of CS under varying levels of diolefinic dye inhibition. Accordingly, Due to the dye's ability to dissolve chloride ions, the diameters of the circle's halves steadily increase when dye concentrations are increased. With the formula below, determine the capacitance of double layers:

8 $${\text{C}}_{{{\text{dl}}}} = {\text{ Y}}_{{\text{o}}} \left( {{2 }\pi {\text{ f}}_{{{\text{max}}}} } \right)^{{{\text{n}} - {1}}}$$

**Fig. 11:**
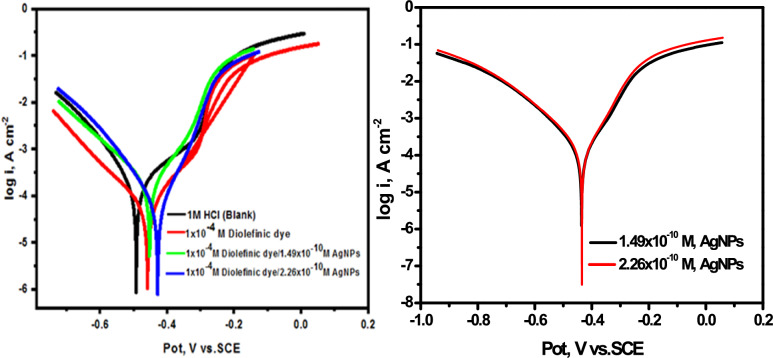
Potentiodynamic polarization curves for corrosion of carbon steel in 1 M HCl in the absence and presence of different concentrations of diolefinic dye with combination of Ag NPs at 298 K

The frequency at which the CPE reaches its greatest value and the measure that shows a departure from the ideal behavior, which ranges from − 1 to 1, respectively, are represented in this example by Y_o_ and f_max_, n. Deviations from a perfect circular shape are often attributed to the phenomenon of interfacial impedance frequency dispersion. This occurrence arises due to various factors, including surface irregularities, impurities, structural defects, grain boundaries, inhibitor adsorption, as well as the formation of porous layers and homogenates on the electrode’s surface [59, 60]. This reduction is attributed to variations in the density of the electrical double layer. “In Fig. 10's Bode phase diagrams, a comprehensive examination of the data reveals that each impedance plot displays a significant capacitive loop characterized by a capacitive time constant”. Using the equivalent circuit depicted within Fig. 11, where R_s_ (the solution resistance), R_ct_, C_dl_, and the constant phase element (CPE) are documented in Table 6**,** the well-fitted data were gathered. “A larger depression is seen in the Nyquist semicircle diagram due to the CPE, which is thought of as an irregular surface irregularity of the electrode and functions as a capacitor at the metal-solution interface”. It is clear that while the C_dl_ values exhibit the opposite association [61], the values of R_ct_ likewise increase as the dye concentration rises. This idea may result from inhibitor adsorption on the metal surface or from H_2_O molecules desorbing from the CS surface [62]. It is evident from increasing the values of n (0.932–0.986, Table 6 for diolefinic dye in comparison to the blank sample that the inhibitor uses adsorption to promote surface uniformity [63]. Additionally, the evaluation of Y_o_ for the reference electrolyte implies that the dye molecules interacted with the electrode surface, thereby mitigating the potential degradation of electrode sites exposed to environmental factors. The corresponding Bode graphs for a CS electrode submerged in 1 M HCl with and without different concentration levels are displayed in Fig. 10. “The only one peak observed in Bode plots manifested the presence of single time constant as mentioned in Nyquist plot”. The unqualified impedance 1Z1 increases at low frequencies, as this figure illustrates. The reason for this rise is the dye's adsorption on the surface of the CS, which blocks its active sites and results in a larger percentage of IE at higher concentrations. Moreover, the negative shift in phase angle values indicates that the main way dye works is by covering the surface of CS with a thin layer [64]. The evaluated values of chi-squared presented in Table 6** “**support good quality of fitting and equivalent circuit used”**.** The data pertaining to R_ct_ serve as a critical component in the computation of the percentage of ionic efficiency (IE) in accordance with the formulation outlined in Eq. (9).

9 $${\text{IE}}_{{{\text{EIS}}}} = {{\varvec{\uptheta}}} \times {1}00 = \frac{{{\text{Rct}}\left( {{\text{inh}}} \right) - {\text{Rct}}}}{{{\text{Rct}}\left( {{\text{inh}}} \right)}} \times {1}00$$

where R_ct(inh)_ and _Rct_ denote the charge transfer resistances in the presence and absence of the inhibitors, respectively.

**Table 6:** EIS data of carbon steel in 1 M HCl and in the absence and presence of different concentrations of diolefinic dye and with combination of Ag NPs at 298 K

Inh	Conc., M	n	Y_o_ µΩ s^n^cm^−2^	C_dl,_ µ F cm^−2^	R_ct_, Ω cm^2^	Θ	%IE	χ^2^
Diolefinic dye	Blank	0.901	215	210	43.27	–	–	19.31 × 10^–3^
	10^–6^	0.932	181	176	95.39	0.540	54.0	22.25 × 10^–3^
	5 × 10^–6^	0.954	176	172	151.03	0.710	71.0	21.18 × 10^–3^
	1 × 10^–5^	0.979	147	143	199.69	0.780	78.0	19.77 × 10^–3^
	5 × 10^–5^	0.983	139	131	240.36	0.810	81.0	20.25 × 10^–3^
	1 × 10^–4^	0.986	125	115	262.78	0.830	83.0	210.33 × 10^–3^

### Synergistic effect

As previously mentioned, the study of synergistic effects can effectively enhance the adsorption uniformity and stability of corrosion inhibitors on metal surfaces, providing a viable solution to the issues. Figure 11 presents the PDP curves for CS in 1 M HCl with varying concentrations of AgNPs. The diagrams indicate that the addition of diolefinic dye (1 × 10^–4^ M) and AgNPs (1.49 × 10^–10^ M or 2.26 × 10^–10^ M) significantly altered the E_corr_ compared to the blank solution. The change in E_corr_ for the HCl and diolefinic dye system was − 34 mV/Ag/AgCl (Table 5), while the changes for the dye with AgNPs were − 41 mV and − 77 mV/Ag/AgCl with the addition of 1.49 × 10^–10^ M and 2.26 × 10^–10^ M AgNPs, respectively.

Table 7 demonstrates that i_corr_ was significantly reduced upon the addition of AgNPs to the dye solutions, and % IE increased from 82 to 85% and 93% with the addition of 1.49 × 10^–10^ M and 2.26 × 10^–10^ M AgNPs, respectively. The high corrosion inhibition efficiencies of the solutions containing AgNPs are attributed to the strong chemisorption of silver ions on metal surfaces. Consequently, diolefinic molecules are adsorbed on the metal surface through Coulombic attractions. The stabilization of the adsorbed Ag ions with the dye results in increased surface coverage and thus greater inhibition effects.

**Table 7:** Corrosion potential (E_corr_), corrosion current density (i_corr_), Tafel slopes (β_c_, β_a_), degree of surface coverage (θ), and inhibition efficiency (IE) of carbon steel in 1 M HCl at 298 K and in the presence of diolefinic dye (1 × 10^–4^ M) and with combination of Ag NPs

Inh	Conc, M	− E_corr_ mV vs. Ag/AgCl	i_corr_ μA cm^−2^	β_a_ mV dec^−1^	− β_c_ mV dec^−1^	k_corr_ mpy	ϴ	% IE
AgNPs	1.49 × 10^–10^	434	148.7	115.16	122.68	1.74	0.34	34
	2.26 × 10^–10^	435	111.5	89.83	124.29	1.308	0.50	50
Diolefinc dye + AgNPs	1 × 10^–4^ M (Dye) + 1.49 × 10^–10^ (AgNPs)	451	35.35	49.337	60.13	0.414	0.85	85.0
	1 × 10^–4^ M (Dye) + 2.26 × 10^–10^ (AgNPs)	415	16.43	42.34	44.23	0.337	0.93	93.0

**Fig. 12:**
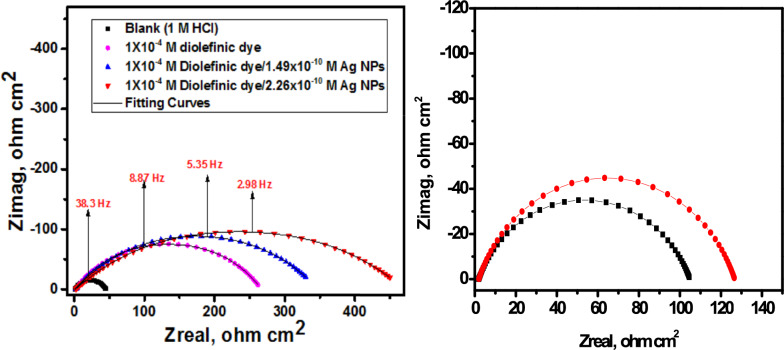
The Nyquist plots for the corrosion of carbon steel in 1 M HCl in the absence and presence of different concentrations of diolefinic dye with combination of Ag NPs at 298

**Fig. 13:**
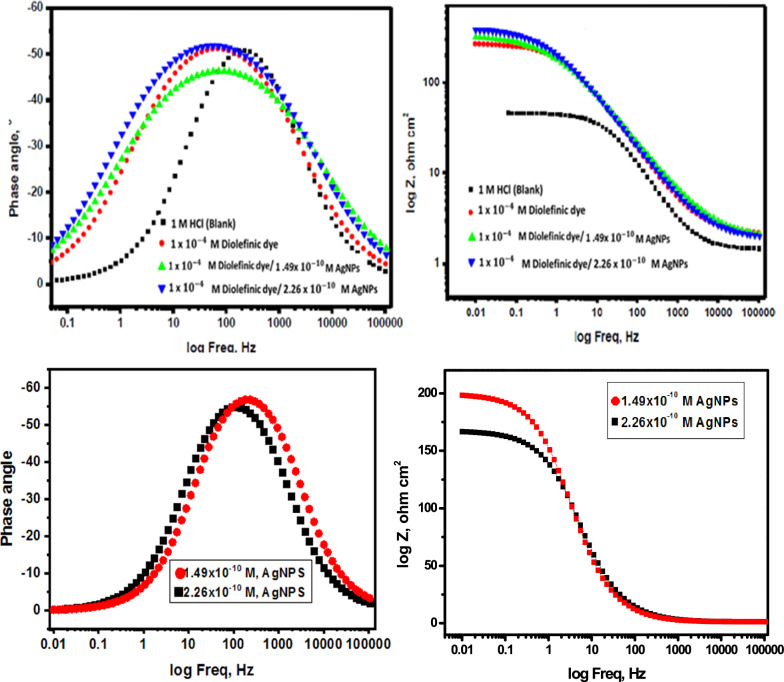
The Bode plots for the corrosion of carbon steel in 1 M HCl in the absence and presence of different concentrations of diolefinic dye with combination of Ag NPs at 298 K

The effect of AgNPs on the % IE of the synthesized dye was observed using EIS. Figures 12 and 13 display the Nyquist impedance and Bode phase diagrams for CS in a corrosive environment with diolefinic dye and AgNPs. The addition of AgNPs to the solution enhanced the protective capability of the dye, as evidenced by the capacitive loop in the diagrams compared to diolefinic dye alone. The corrosion rate decreased significantly with the addition of AgNPs (Table 8). Beyond this concentration, the corrosion rate decreased gradually, attributed to the increased surface coverage (θ) of the dye molecules on metal surface with increasing concentration. The corrosion % IE also increased with higher concentrations of AgNPs (1.49 × 10^–10^ M or 2.26 × 10^–10^ M). With a dye concentration of 1 × 10^–4^ M, the maximum IE was 83% (Table 6), indicating that a single dye cannot effectively protect CS from corrosion in HCl. This value increased to 87% and 90% with the addition of 1.49 × 10^–10^ M and 2.26 × 10^–10^ M AgNPs, respectively (Table 8).

**Table 8:** EIS data of carbon steel in 1 M HCl and in the presence of diolefinic dye (1 × 10^–4^ M) and with combination of Ag NPs at 298 K

Inh	Conc, M	R_ct_, Ω cm^2^	Θ	%IE
AgNPs	1.49 × 10^–10^	104	0.48	48.0
	2.26 × 10^–10^	124	0.65	65.0
Diolefinc dye + AgNPs	1 × 10^–4^ M (Dye) + 1.49 × 10^–10^ (AgNPs)	331.3	0.870	87.0
	1 × 10^–4^ M (Dye) + 2.26 × 10^–10^ (AgNPs)	450.0	0.900	90.0

The synergism parameter (SI) was evaluated using the relationship provided by Aramaki and Hackerman, as reported in previous studies  (Eq. 10) [65, 66]:

10 $${\text{SI}} = \frac{{1 - {\text{I}}_{1 + 2} }}{{1 - {\text{I}}_{1 + 2}^{ - } }}$$

where I_1+2_ = I_1_ + I_2_; I_1_, I_2_ are the inhibition efficiencies of the AgNPs and the diolefinic dye, and I′ is the measured inhibition efficiency for diolefinic dye in combination with AgNPs. This parameter was determined from inhibition efficiency values obtained through PDP and EIS measurements. The results, presented in Table 9**,** were found to be greater than unity, indicating strong inhibition effects on CS due to the synergistic effect of AgNPs, which initially adsorb onto the metal surface, followed by inhibitor cations.

**Table 9:** Synergistic parameters (SI) of the inhibitor diolefinic dye with combination of AgNPs

Inhibitor	Synergism parameter (SI)		
	Conc, M	Polarization	EIS
Diolefinc dye + AgNPs	1 × 10^–4^ M (Dye) + 1.49 × 10^–10^ (AgNPs)	1.369	1.511
	1 × 10^–4^ M (Dye) + 2.26 × 10^–10^ (AgNPs)	1.423	1.651

Reports suggest that AgNPs can interact with the metal surface and decrease its hydrophobicity [67, 68]. AgNPs may chemisorb onto the CS surface in addition to the physisorption of cationic molecules. On the metal surface, AgNPs interact with the metal surface and diminish the repulsive effects exerted by the metal surface on the adsorbed layer.

### Surface examinations

#### SEM analysis

**Fig. 14:**
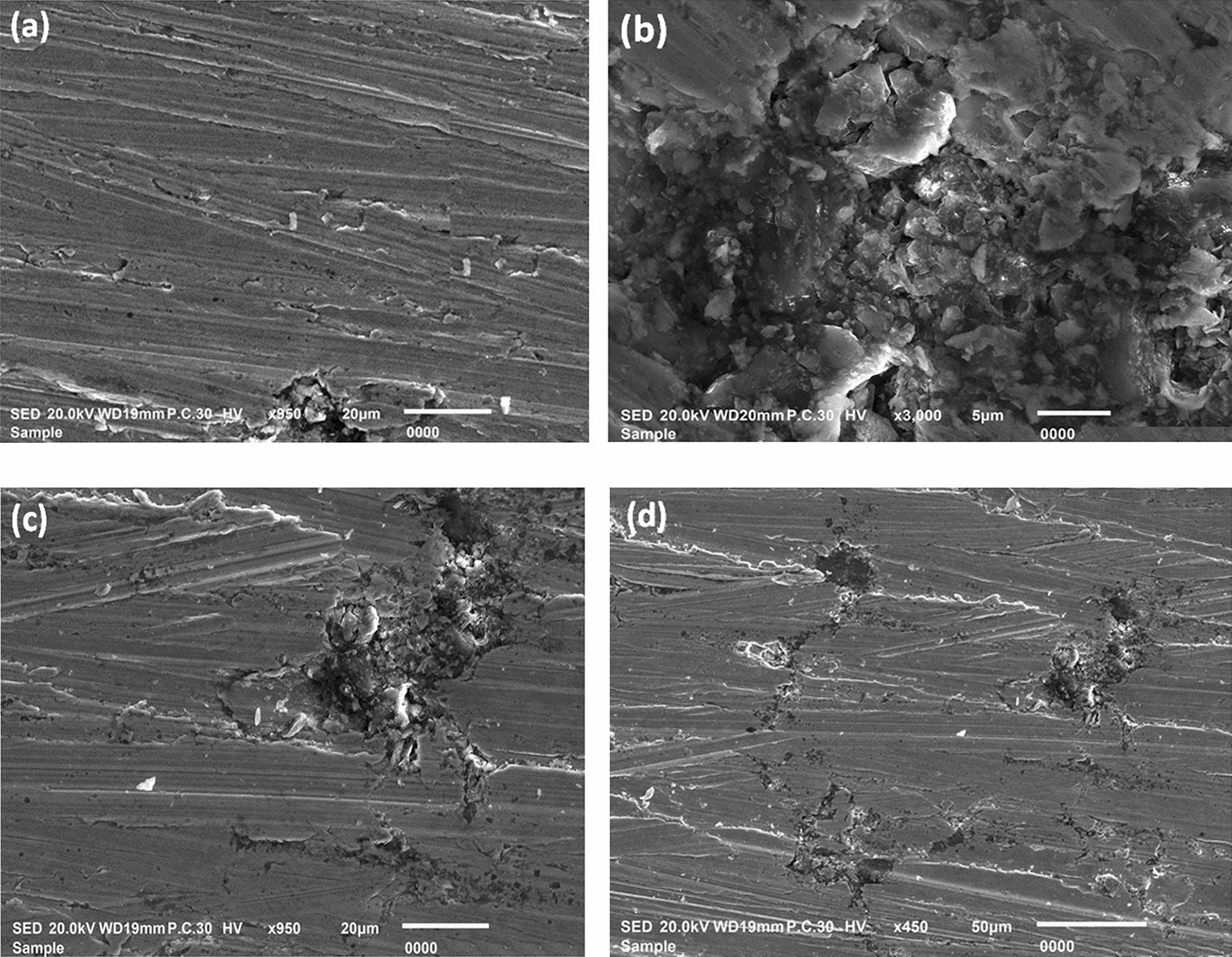
SEM micrographs of carbon steel surface: **a** before immersion in 1.0 M HCl, **b** after 24 h of immersion in 1.0 M HCl, **c** after 24 h of immersion in 1.0MHCl + 5 × 10^−5^ M diolefinic dye, and **d** after 24 h of immersion in 1.0 M HCl + 5 × 10^−5^ M diolefinic dye in the presence of 0.01 M AgNPs at 298 K

The CS specimens underwent SEM analysis after their immersion in a hydrochloric acid (HCl) solution of one molar concentration, both under inhibitor-free and inhibitor-containing conditions, while being maintained at a temperature of 25 °C, as illustrated in Fig. 14. In Fig. 14a, the microstructure of the steel is readily distinguishable, exhibiting discernible grain boundaries comprised of ferrite and pearlite [9, 69]. However, prolonged exposure to the 1.0 M HCl solution for duration of 24 h resulted in the complete degradation of the steel's microstructure, leading to the formation of significant pits, as shown in Fig. 14b. Conversely, the incorporation of inhibitor molecules yielded a notable reduction in steel damage, resulting in complete surface coverage when the diolefinic dye was present, as demonstrated in Fig. 14c. Subsequent SEM test of the CS surface after being exposed to a solution comprising one molar HCl, 5 × 10^–5^ M diolefinic dye, and 2.26 × 10^–10^ M AgNPs for duration of 24 h is depicted in Fig. 14d. The synergistic impact of the dye and AgNPs has yielded a remarkably smoother metal surface, indicative of an augmented inhibitory effect. These findings are consistent with prior empirical observations that have underscored the superior inhibitory efficacy of diolefinic compounds when combined with AgNPs.

#### EDX analysis

**Fig. 15:**
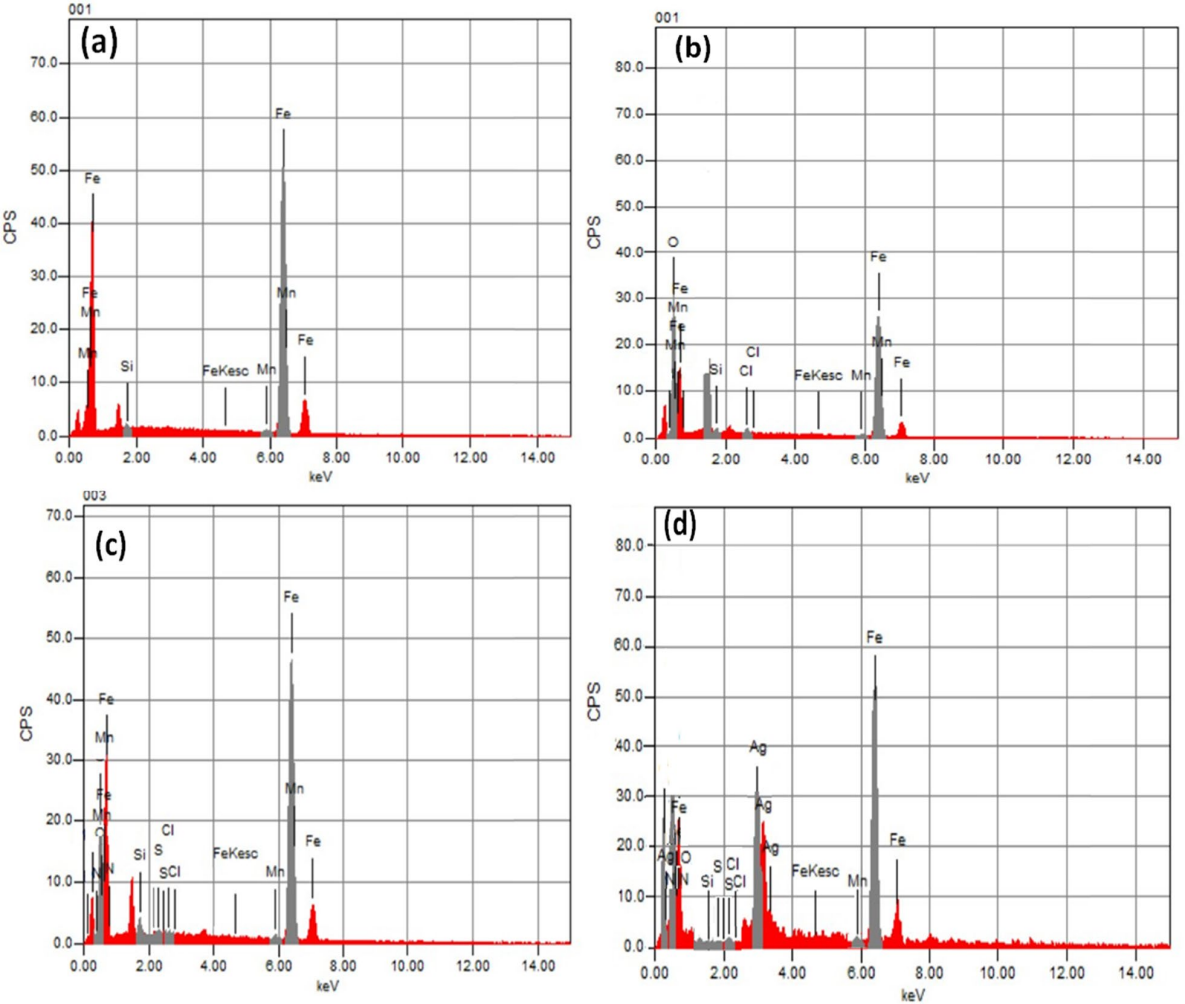
EDX spectra of carbon steel specimens of **a** before immersion in 1.0 M HCl, **b** after 24 h of immersion in 1.0 M HCl, **c** after 24 h of immersion in 1.0MHCl + 5 × 10^−5^ M diolefinic dye, and **d** after 24 h of immersion in 1.0 M HCl + 5 × 10^−5^ M diolefinic dye in the presence of 0.01 M AgNPs at 298 K

The EDX spectra, as illustrated in Fig. 15a & b, depict the CS surface's elemental composition under differing conditions: one devoid of any treatment and the other immersed in one molar HCl solution. Figure 15c portrays the EDX spectra of the CS surface subjected to a one molar HCl solution enriched with 5 × 10^–5^ M diolefinic dye, while Fig. 15d displays spectra of the same sample dipped in a solution containing both 5 × 10^–5^ M diolefinic dye and 2.26 × 10^–10^ M AgNPs. The EDX analysis results, tabulated in Table 10**,** elucidate the atomic percentages of various elements on the CS surface in its pristine state, without inhibition, and with inhibition measures applied. Specifically, in the case of a CS specimen immersed in a 1.0 M HCl solution, the atomic percentage of iron registers at 72.72%. This value rises to 80.35% when the specimen is exposed to a solution containing 5 × 10^–5^ M diolefinic dye and further elevates to 83.10% when the solution includes 2.26 × 10^–10^ M AgNPs. The discernible presence of relatively smoother iron peaks in EDX spectra of samples containing a concentration of 2.26 × 10^–10^ M AgNPs, when juxtaposed with those of a meticulously polished sample immersed in a dye solution, implies the development of a more substantial and effective corrosion-inhibiting coating on the surface of the test specimen. Furthermore, the EDX spectra of the inhibited samples manifest the discernible emergence of peaks corresponding to all constituent elements of the dye molecules, thereby substantiating the process of dye adsorption onto the surface of the specimen.

**Table 10:** Percentage atomic contents of elements obtained from EDX spectra

Inhibitors	Fe	Si	Mn	O	Cl	S	N	Ag
Carbon steel (Free)	97.82	0.88	1.31	–	–	–	–	–
Carbon steel in 1.0 M HCl	72.72	0.38	0.41	25.99	0.50	–	–	–
Carbon steel in 1 × 10^–5^ M diolefinic dye + 1 M HCl	80.35	1.29	0.67	15.29	0.17	0.28	1.95	–
Carbon steel in 1 × 10^–5^ M diolefinic dye + 2.26 × 10^–10^ AgNO_3_ + 1 M HCl	83.10	0.31	0.53	3.02	0.13	0.19	1.25	11.47

#### AFM analysis

**Fig. 16:**
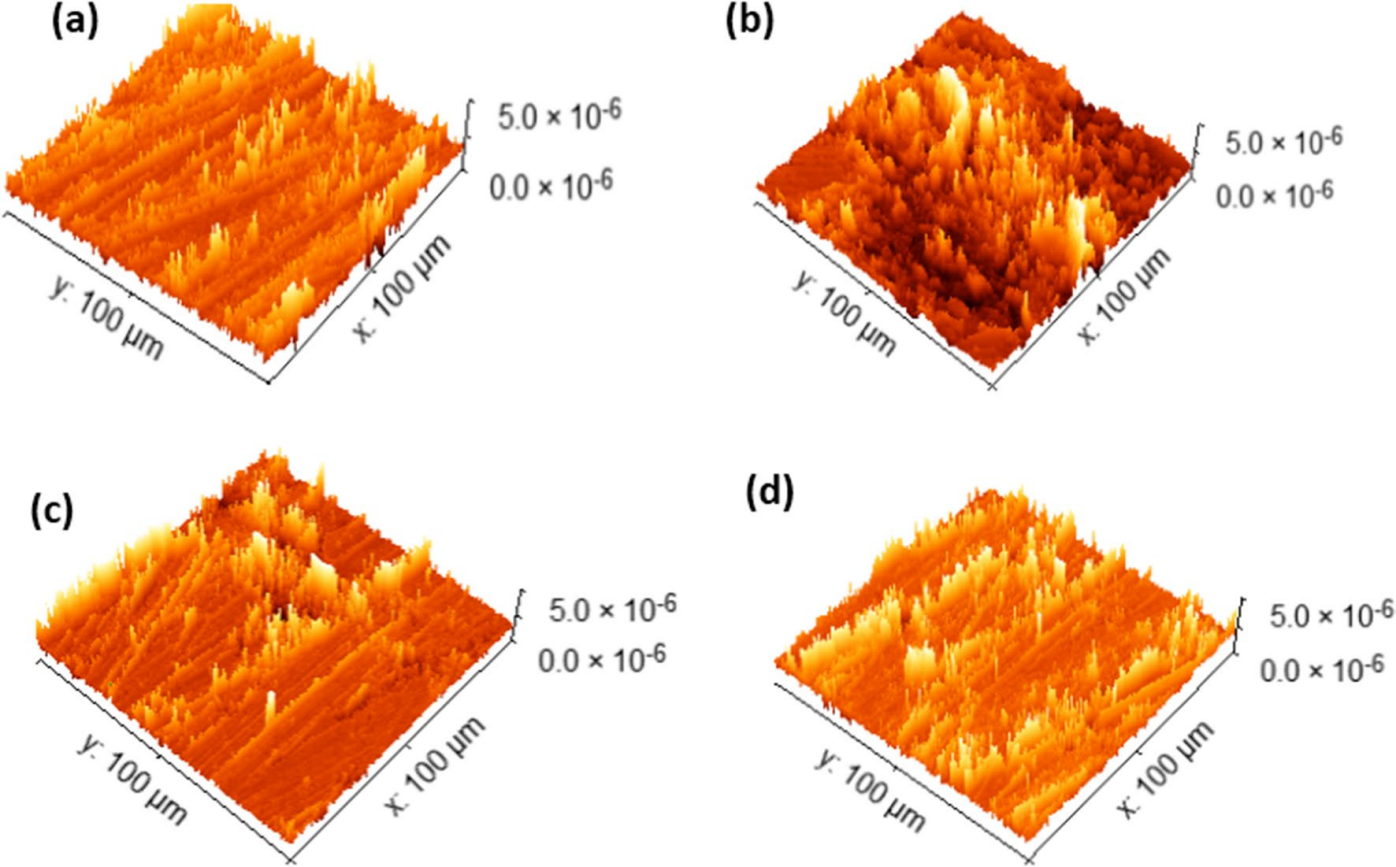
AFM images of the carbon steel surface **a** before immersion in 1.0 M HCl, **b** after 24 h of immersion in 1.0 M HCl, **c** after 24 h of immersion in 1.0MHCl + 5 × 10^−5^ M diolefinic dye, and **d** after 24 h of immersion in 1.0 M HCl + 20 × 10^−5^ M diolefinic dye in the presence of 0.01 M AgNPs at 298 K

To augment the substantiation of the adsorption mechanism exhibited by inhibitor molecules adhering to the metal substrate, a comprehensive methodology encompassing the utilization of AFM and SEM was deployed to scrutinize the surface topography of CS across different scenarios, namely, in the lack of an inhibitor and in the presence of the most effective inhibitor concentrations. The resultant findings are succinctly presented in Table 11, elucidating pertinent surface roughness parameters, including the arithmetic mean roughness (R_a_), root mean square roughness (R_q_), and dipping peak height (R_p_). Figure 16a–d presents three-dimensional AFM images depicting the CS surface under various experimental conditions, “including its initial state before introduction to corrosive media, subsequent to a 24-h dipping in a one molar HCl solution, following a 24-h dipping in a one molar HCl solution enriched with a higher concentration of 5 × 10^–5^ M diolefinic dye, and after a 24-h dipping in a one molar HCl solution containing both 5 × 10^–5^ M dye and 2.26 × 10^–10^ M AgNPs. The outcomes illustrate significant discrepancies in the state of the CS surface. Following a 24-h dipping in a one molar HCl solution, it was observed that the CS surface exhibited significant corrosion and structural deterioration in comparison to the unaltered pristine CS surface. This observation is substantiated by the notable increase in surface roughness parameters, specifically R_q_, R_a_, and R_max_, as illustrated in Fig. 16b. Conversely, when the metal surface was subjected to a 24-h immersion in one molar HCl solution containing a higher concentration of 5 × 10^–5^ M diolefinic dye, there was a discernible reduction in structural disruption, resulting in a smoother surface topography when compared to the sample immersed in the acidic solution devoid of the dye. This phenomenon is supported by the lower values of surface roughness parameters, as depicted in Fig. 16c. Notably, following a 24-h exposure period within one molar HCl solution comprising both a concentration of 5 × 10^–5^ M dye and 2.26 × 10^–10^ M AgNPs, the surface of CS exhibited a marked improvement in smoothness, as evidenced by significantly reduced surface roughness values. This phenomenon strongly implies an enhanced inhibitory mechanism attributable to the cooperative effects of the dye and AgNPs, as visually represented in Fig. 16d.

**Table 11:** Roughness values obtained from AFM for the carbon steel surface in 1.0 M HCl with and without diolefinic dye and diolefinic dye + AgNPs

Inhibitors	*R*_a_ (nm)	*R*_q_ (nm)	*R*_p_ (nm)
Carbon steel (free)	17.05	21.61	45.32
Carbon steel + 1.0 M HCl	78.32	105.2	334.4
Carbon steel + 1.0 M HCl + Diolefinic dye	50.13	69.32	201.1
Carbon steel + 1.0 M HCl + Diolefinic dye + AgNPs	31.03	38.83	79.57

### Theoretical studies

#### Quantum chemical calculations

**Fig. 17:**
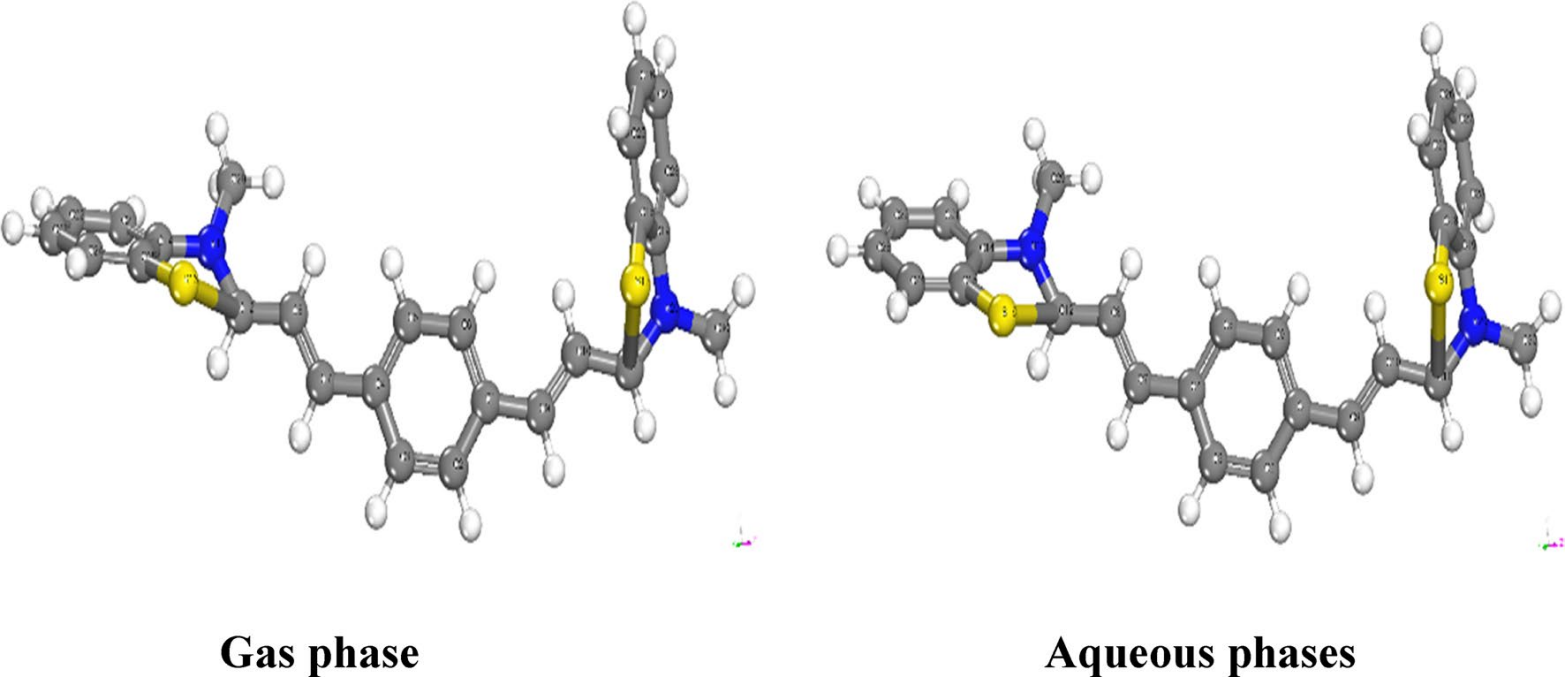
The optimized molecular structures of the investigated inhibitor, diolefinic dye in gas and aqueous phases

The experimental investigation focused on assessing the corrosion inhibition properties of the synthesized inhibitor on CS. Furthermore, a series of quantum chemical simulations were conducted to analyze how various structural factors impact the efficacy of the inhibitor, as well as to understand the adsorption processes occurring on the metal surface. The molecular structures of the inhibitors were systematically optimized by calculating bond lengths and bond angles, as presented in Tables S1& S2 in the Supplementary Materials. The optimized molecular structures, obtained from both gas and water phase calculations, are depicted in Fig. 17. Quantum chemical parameters” essential for assessing the inhibitor's effectiveness, such as parameter B, were derived from these calculations and are provided in Table 12. This includes the energies of the highest occupied molecular orbital (E_HOMO_), the energies of the lowest unoccupied molecular orbital (E_LUMO_), and the energy gap (E) between them, which serves as a measure of reactivity, electronegativity (χ), dipole moment (D), softness (σ), chemical potential (μ), and hardness (η). In accordance with Koopmans' theorem [70], the E_HOMO_ and E_LUMO_ values of the inhibitor molecules are closely associated with their electron affinity (A) and ionization potential (I), respectively. Moreover, additional quantum chemical properties were derived from specific relationships to provide crucial insights into the reactive activity of the inhibitors [71].

11 $$\upchi \left(\text{electronegativity}\right)=\frac{-({E}_{LUMO} + {E}_{HOMO})}{2} $$

12 $$\upmu (\text{potential}) = -\upchi = \frac{\left({E}_{LUMO} + {E}_{HOMO}\right)}{2} $$

13 $$\upeta (\text{hardness}) = \frac{({E}_{LUMO}- {E}_{HOMO})}{2} $$

14 $$\omega \, \left( {{\text{electrophilicity}}} \right) \, = \, \mu^{{2}} /{2}\eta $$

**Table 12:** The calculated quantum chemical parameters obtained from Dmol^3^ calculations in gas and aqueous phases

Inhibitor	E_HOMO_ (eV)	E_LUMO_ (eV)	ΔE (eV)	D (Debye)	η (eV)	σ (eV^−1^)	μ (eV)	χ (eV)	ω (eV)	∆*N*
Gas phase	− 3.552	− 2.559	0.993	4.2827	0.497	2.014	− 3.06	3.06	9.40189	3.972
Aqueous phases	− 3.690	− 2.679	1.011	8.0445	0.506	1.978	− 3.18	3.18	10.0307	3.774

“The softness is defined as the inverse of the global hardness, which is as follows”:

15 $$\sigma \, \left( {{\text{softness}}} \right) \, = {1}/\eta $$

“The fraction of electrons (ΔN) exchanged between the inhibitor and the metallic surface is calculated as” [72, 73]:

16 $$\Delta \text{N}=\frac{({\upchi }_{\text{Fe}}-{\upchi }_{\text{inh}})}{2({\upeta }_{\text{Fe}}+{\upeta }_{\text{inh}})} $$

where a theoretical value of χFe ≈ 7 eV and ηFe = 0 is taken based on the assumption that I = A for a bulk metal because they are softer than neutral metallic atoms. Within the framework of frontier orbital theory, the manifestation of chemical reactivity is predicated upon the intricate interplay “between the Highest Occupied Molecular Orbital (HOMO) and the Lowest Unoccupied Molecular Orbital (LUMO)” energy levels of the chemical entities involved [74]. In the context of vacant molecular orbitals, the EHOMO denotes the molecule's inherent ability to contribute electrons to a compatible electron-accepting entity, whereas the ELUMO signifies its inclination to receive electrons. A diminished ELUMO value corresponds to an augmented capacity for electron acceptance [75]^.^ For inhibitors, a higher E_HOMO_ (Highest Occupied Molecular Orbital Energy) value serves to facilitate the transfer of electrons to metal surfaces, thereby augmenting the inhibitory potential. Specifically, the E_HOMO_ values for the examined inhibitor in gaseous and aqueous environments are recorded as − 3.552 eV and − 3.690 eV, respectively. These values elucidate the propensity of the inhibitor to adsorb onto metal surfaces and elucidate its inhibitory characteristics, as presented in Table 13. The energy gap, denoted as ΔE_gap_ and defined as the difference between E_HOMO_ and E_LUMO_ (Lowest Unoccupied Molecular Orbital Energy), stands as a critical parameter employed for assessing stability and constructing theoretical models to elucidate structural attributes and conformational barriers in diverse molecular systems. A lower ΔE_gap_ value signifies a more efficacious inhibitor. As per our calculations, the inhibitor exhibits a narrow energy gap in both gaseous (0.993 eV) and aqueous (1.011 eV) phases, underscoring its inclination towards binding with metal surfaces. The absolute hardness (η) and softness (σ) of a molecule play a pivotal role in determining its stability and reactivity. A high energy gap characterizes hard molecules, whereas soft molecules exhibit a small energy gap. The enhanced reactivity of soft molecules stems from their facile electron donation to acceptors. Adsorption predominantly occurs in the molecular region with the highest σ value, a local attribute [76] In a corrosion system, the metal functions as a Lewis acid, while the inhibitor acts as a Lewis base. Given that bulk metals qualify as soft acids, soft base inhibitors prove especially efficacious against acid corrosion of these metals. Consequently, the σ values in the gas and water phases (2.014 and 1.978 eV^−1^, respectively) demonstrate the inhibitory prowess of the inhibitors. Furthermore, computational analysis revealed that the inhibitor possesses χ values (3.06 and 3.18 eV) in both phases, elucidating its capacity for donating electrons to the metal surface. When ΔN exhibits a positive value, the process of electron transfer is observed from the inhibitor to the metal. Conversely, when ΔN takes a negative value, electron transfer occurs from the metal to the inhibitor. Furthermore, Lukovits et al. [77] demonstrated that when ΔN is less than 3.6, there exists a correlation between inhibitory potency and the capacity to donate electrons to the metal, thereby enhancing the augmentation of the metal surface. The data presented in Table 13 indicate that all ΔN values remain positive across both phases, suggesting that the inhibitor can effectively contribute electrons to the iron surface, facilitating the formation of self-assembled layers of the inhibitor. However, the ΔN value for the inhibitor slightly surpasses 3.6, signifying a diminishing inhibitory impact of the inhibitor in conjunction with its electron-donating capacity to the metal surface. Moreover, as depicted in Fig. 18, the highest occupied molecular orbital (HOMO) level of all the inhibitors predominantly localizes to the central aromatic ring. This observation strongly suggests that the ring represents the favored site for electrophilic interaction with the metal surface. It follows that aromatic rings with elevated HOMO density coefficients are positioned toward the metal surface, indicating that adsorption is primarily facilitated by the π-electrons of these aromatic rings. Additionally, the computational results demonstrate that the charge density distribution within the lowest unoccupied molecular orbital (LUMO) planes is extensively spread across the central aromatic ring. This finding implies that this molecular component has the potential to function as an electrophile (electron acceptor).

**Table 13:** Outputs and descriptors calculated by the Monte Carlo simulation for adsorption of the inhibitor on Fe (110) surface with increasing if inhibitor monomer numbers

# Inhibitor monomers	Total energy (Kcal/mol)	Adsorption energy (Kcal/mol)	Binding energy (Kcal/mol)	Rigid adsorption energy (Kcal/mol)	Deformation energy (Kcal/mol)	Inhibitor: dE_ad_/dNi
1	− 226.52	− 224.61	224.61	− 241.47	16.86	− 224.61
2	− 453.48	− 449.67	449.67	− 483.32	33.64	− 226.54
3	− 653.59	− 647.87	647.87	− 709.23	61.36	− 215.93
4	− 805.36	− 797.74	797.74	− 861.84	64.10	− 178.36
5	− 873.28	− 863.75	863.75	− 957.14	93.40	− 131.95
6	− 990.06	− 978.62	978.62	− 1046.90	68.28	− 128.12
7	− 1062.18	− 1048.83	1048.83	− 1150.45	101.62	− 138.90
8	− 1108.50	− 1093.24	1093.24	− 1194.32	101.07	− 81.39

**Fig. 18:**
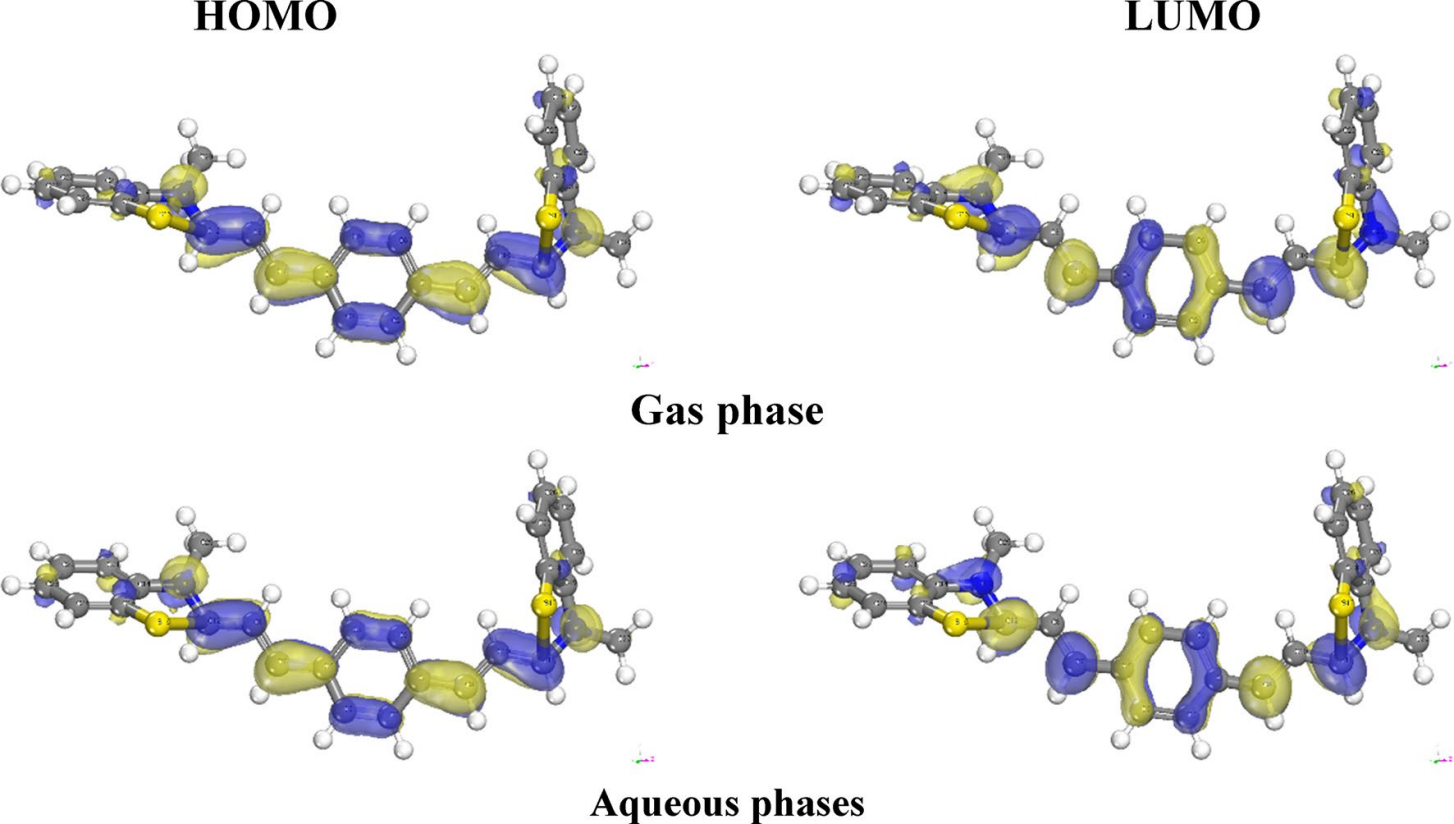
The highest occupied molecular orbital and the lowest unoccupied molecular orbital of the investigated diolefinic dye

#### Molecular electrostatic potentials (MEPs)

**Fig. 19:**
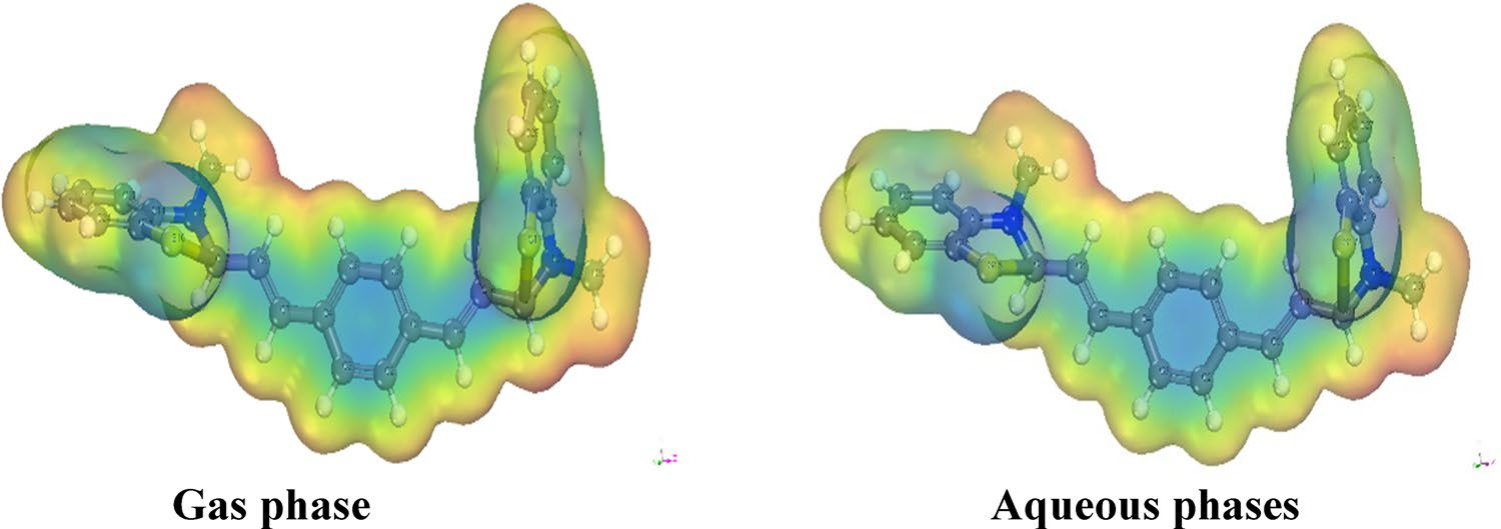
The molecular electrostatic potentials of optimized structures of the investigated diolefinic dye

The MEPs, as illustrated in Fig. 19, hold significant importance due to their ability to identify regions with negative electrostatic potential, which can be regarded as nucleophilic centers. Conversely, regions with positive electrostatic potential can be deemed as potential electrophilic sites. Furthermore, the electrostatic potential serves as an indicator of electron density polarization. In a descending sequence, the distribution of electric density can be delineated as follows: “red > orange > yellow > green > blue”. The regions exhibiting negativity, (depicted in red) manifest electrophilic traits, while the positively charged regions (displayed in blue) denote a dearth of electrons. Upon meticulous scrutiny of the visual representation, it becomes evident that the zones abundant in electron density (signified by the red hue) showcase a state of electron delocalization within locales encompassing nitrogen atoms (N13 and N_2_0) and their adjacent atomic counterparts. In contrast, the remaining locales are typified by a diminished electron density. These locales marked by heightened electron density foster an electronic interaction with unoccupied iron d orbitals, thereby engendering a propensity for spontaneous adsorption between the inhibitor molecule and the iron surface [78, 79].

#### Molecular dynamic simulations

##### Effect of diolefinic concentration

**Fig. 20:**
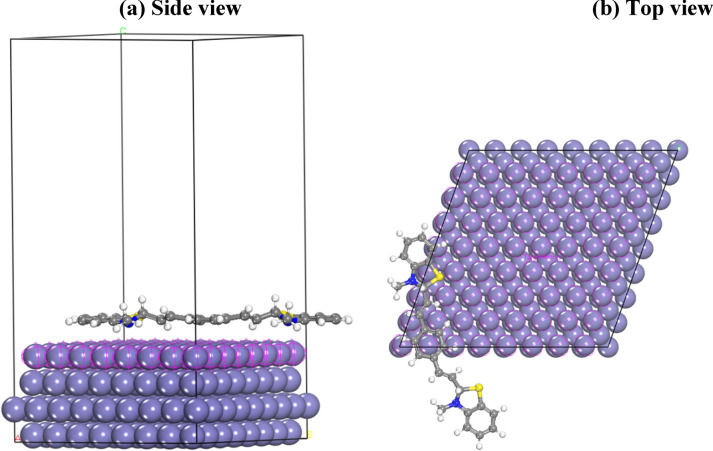
Molecular simulations for the most favorable modes of adsorption obtained for the inhibitor on Fe (110) surface, side view (**a**) and top view (**b**)

The examined inhibitor exhibits distinct active places for adsorption onto the metal surface. Considering this, a molecular dynamics simulation was conducted, involving the inhibitor under examination and the iron surface, to ascertain the most favorable adsorption site for the communication between the inhibitor compounds and the Fe (1 1 0) surface. The geometric configurations of the adsorbate constituents were optimized until they satisfied specific criteria. Figure 20 illustrates the lateral and top perspectives of the modeling of inhibitor adsorption onto the Fe (1 1 0) surface. “According to the visual representation”, the inhibitor can adhere to the iron surface through the charge distribution on the central aromatic ring and the lone electron pairs associated with nitrogen atoms. These interactions facilitate the formation of coordinated bonds between the amino acid complex and the iron surface, resulting in the creation of a densely packed layer that hinders the movement of corrosive agents towards the metal surface. The subsequent relationship was employed for the control of the contact energy (commonly referred to as binding energy) between the inhibitor molecule and the iron surface:

17 $${\text{E}}_{{{\text{ads}}}} = {\text{E}}_{{{\text{Fe}}}} - {\text{inh }} - \left( {{\text{E}}_{{{\text{inh}}}} + {\text{E}}_{{{\text{Fe}}}} } \right) $$

**Fig. 21:**
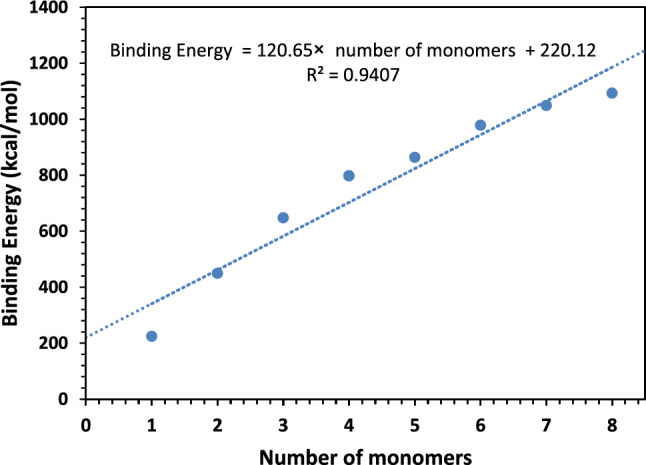
The binding energies according to the number of monomers of the inhibitor on the Fe (110) surface

The total energies of the inhibitor (E_inh_) and the Fe surface (E_Fe_) were determined, leading to the calculation of a notably extensive binding energy (E_bind_ = − E_ads_ = 224.61 kcal/mol). This negative adsorption energy value elucidates the inhibitor molecule's spontaneous adsorption onto the metal surface and underscores the remarkably high level of inhibition effectiveness [80, 81] Table 14 displays the binding and adsorption energies achieved through the incremental introduction of inhibitor monomers up to a count of eight. Nevertheless, surpassing this monomer count led to a termination of the molecular dynamics (MD) interaction simulation. The ascending trend of adsorption energy with escalating monomer concentration aligns with empirical observations. This trend potentially relates to an augmentation in the functional groups within the studied inhibitor monomer, implying heightened stability and the intrinsic tendency of the resulting complex. Figure 21 visually demonstrates the strong correlation between the MD outcomes and the experimental data.

**Table 14:** Outputs and descriptors calculated by the Monte Carlo simulation for adsorption of the inhibitor on Fe (1 1 0) surface in absence and presence of AgNPs

Compound	Total energy (Kcal/mol)	Adsorption energy (Kcal/mol)	Binging energy (Kcal/mol)	Rigid adsorption energy (Kcal/mol)	Deformation energy (Kcal/mol)	Inhibitor: dEad/dNi	H_3_O^+^: dEad/dNi	HCl: dEad/dNi	H_2_O: dEad/dNi	Ag: dEad/dNi
Inhibitor	− 1082.34	− 1080.43	1080.43	− 1116.73	36.31	− 230.69	− 117.13	− 4.08	− 11.19	–
Inhibitor + Ag	− 1126.77	− 1112.93	1112.93	− 1142.02	29.09	− 210.84	− 88.95	− 15.25	− 11.22	− 208.82

##### Effect of AgNPs

**Fig. 22:**
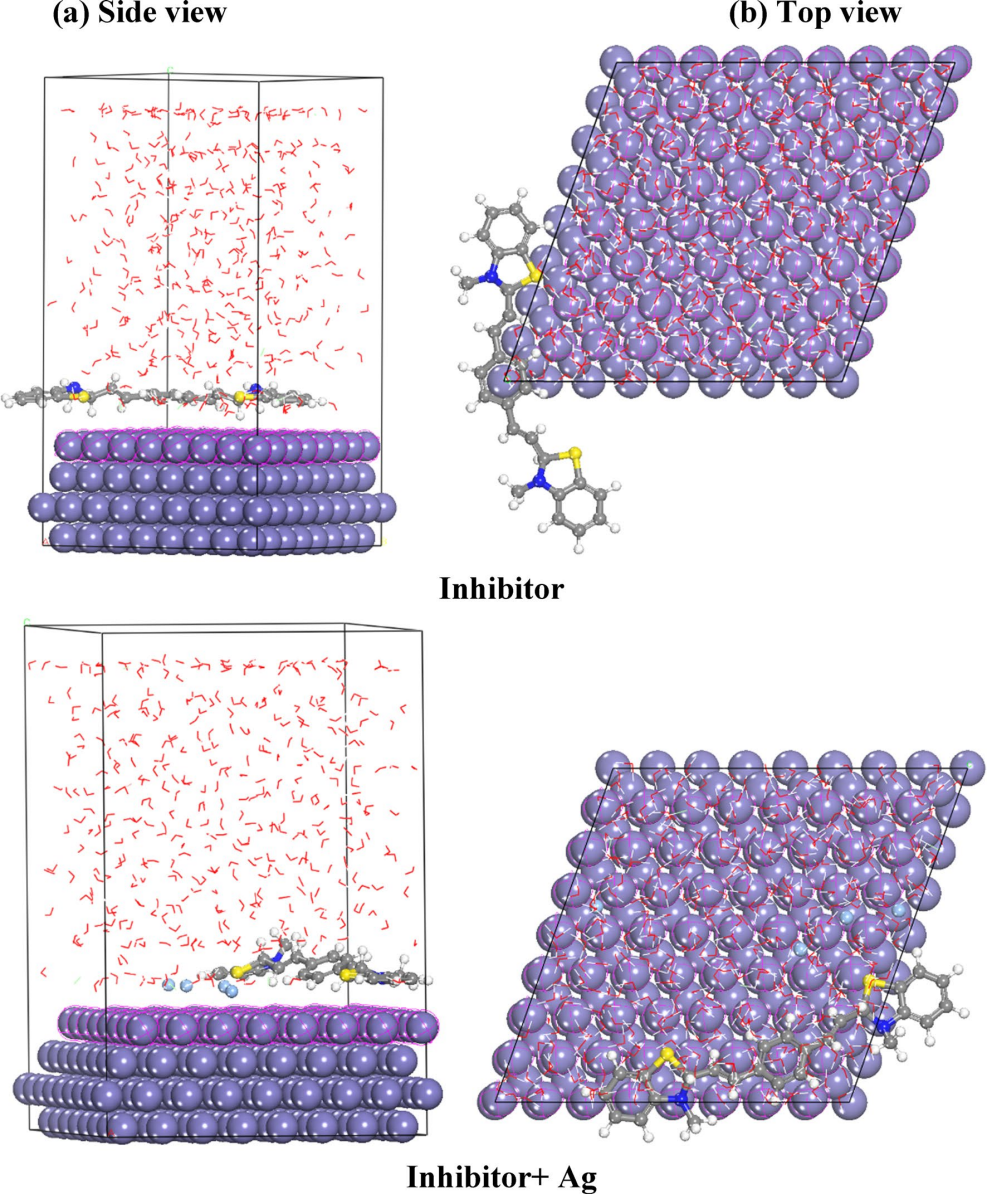
Molecular simulations for the most favorable modes of adsorption obtained for the inhibitor in absence and presence of Ag molecules on Fe (110) surface, side view (**a**) and top view (**b**)

A direct relationship emerges between the binding energy magnitude and the facilitation of inhibitor adsorption onto the metal surface, leading to heightened inhibition efficiency. The binding energy, as shown in Table 14, displays higher values when Ag molecules are introduced compared to their absence. This observation aligns with experimental findings demonstrating that the presence of Ag molecules augments the inhibitor's inhibition efficiency. The configuration of optimal adsorption, both without and with Ag molecules, onto the Fe (1 1 0) surface is illustrated in Fig. 22. A detailed analysis of Fig. 21 (Top view) reveals an expanded coverage of the Fe (1 1 0) surface by the dye in the presence of Ag molecules, potentially attributing to the detected elevation in % IE.

## Conclusions

The synergistic mechanism of Diolefinc dye with AgNPs for corrosion inhibition of CS in 1 M HCl was investigated. The main findings from this work are as follows:

1. Diolefinic dye findings indicate that the investigated dye serves as effective corrosion inhibitor for CS in 1 M HCl solution.
2. % IE increases on increasing the dose of the dye and with increasing temperature.
3. The temperature effect shows that Diolefinic dye is chemisorbed on the CS surface.
4. Adsorption of the dye obeyed Langmuir adsorption isotherm model.
5. The synergy coefficient (SI) of Diolefinc dye and AgNPs were greater than unity for all concentration conditions, confirming the existence of good synergy between Diolefinc dye and AgNPs.
6. The EIS data showed that Diolefinc dye and AgNPs have a certain corrosion inhibition effect on CS in HCl solution. The maximum % IE was 90%, 65% and when Diolefinc dye and AgNPs concentrations were 1 × 10^–4^, 2.26 × 10^–10^ M, respectively.
7. PDP studies suggested that the dye acts as` a mixed-kind inhibitor.
8. SEM and AFM confirmed the presence of inhibitor protective film on CS surface.
9. Theoretical results obtained by DFT and m are in good correlation with the experimental results obtained by the WL and electrochemical studies.

## Supplementary Information

**Additional file 1.**

## Data Availability

All data generated or analyzed during this study are included in this published article.
