# Supplementary material for: Synergistic inhibition effect of diolefinic dye and silver nanoparticles for carbon steel corrosion in hydrochloric acid solution
Source: BMC Chem. 2024 Oct 5;18(1):193. doi: 10.1186/s13065-024-01298-w (PMC11456253; doi:10.1186/s13065-024-01298-w)
Supplement: Supplementary file 1 — Additional file 1. [file 13065_2024_1298_MOESM1_ESM.docx]

**Table S1** Selected bond length (Å) of inhibitor in gas and aqueous phases.

| Bond length (Å) | gas phase | aqueous phase | Bond length (Å) | gas phase | aqueous phase |
| --- | --- | --- | --- | --- | --- |
| C(27)-C(28) | 1.419 | 1.42 | N(13)-C(14) | 1.432 | 1.424 |
| C(26)-C(27) | 1.422 | 1.422 | C(12)-S(16) | 3.146 | 3.159 |
| C(25)-C(26) | 1.417 | 1.42 | N(13)-C(12) | 1.393 | 1.399 |
| C(24)-C(23) | 1.412 | 1.42 | N(20)-C(11) | 1.387 | 1.39 |
| C(23)-C(22) | 1.42 | 1.42 | C(11)-S(17) | 3.207 | 3.305 |
| C(22)-C(21) | 1.415 | 1.417 | C(10)-C(11) | 1.411 | 1.414 |
| N(20)-C(30) | 1.474 | 1.476 | C(9)-C(10) | 1.415 | 1.417 |
| C(28)-C(19) | 1.426 | 1.43 | C(8)-C(12) | 1.41 | 1.411 |
| N(20)-C(19) | 1.428 | 1.43 | C(7)-C(8) | 1.416 | 1.418 |
| C(18)-C(25) | 1.435 | 1.435 | C(5)-C(6) | 1.411 | 1.413 |
| C(18)-C(19) | 1.448 | 1.45 | C(4)-C(7) | 1.438 | 1.441 |
| S(17)-C(18) | 1.767 | 1.774 | C(4)-C(5) | 1.44 | 1.444 |
| C(15)-C(24) | 1.434 | 1.435 | C(3)-C(4) | 1.439 | 1.442 |
| S(16)-C(15) | 1.766 | 1.776 | C(2)-C(3) | 1.413 | 1.413 |
| C(21)-C(14) | 1.427 | 1.432 | C(1)-C(9) | 1.439 | 1.441 |
| C(15)-C(14) | 1.451 | 1.451 | C(1)-C(6) | 1.438 | 1.441 |
| N(13)-C(29) | 1.476 | 1.479 | C(1)-C(2) | 1.44 | 1.444 |

**Table S2** Selected bond angle (^o^) of inhibitor in gas and aqueous phases.

| Bond angle (^o^) | gas phase | aqueous phase | Bond angle (^o^) | gas phase | aqueous phase |
| --- | --- | --- | --- | --- | --- |
| C(27)-C(28)-C(19) | 120.469 | 120.583 | C(15)-C(14)-N(13) | 121.381 | 121.083 |
| C(28)-C(27)-C(26) | 119.868 | 119.765 | C(29)-N(13)-C(14) | 117.524 | 118.127 |
| C(27)-C(26)-C(25) | 120.076 | 120.205 | C(29)-N(13)-C(12) | 122.691 | 121.507 |
| C(26)-C(25)-C(18) | 121.329 | 121.276 | C(14)-N(13)-C(12) | 119.613 | 119.575 |
| C(23)-C(24)-C(15) | 121.741 | 121.646 | S(16)-C(12)-N(13) | 78.914 | 78.061 |
| C(24)-C(23)-C(22) | 119.595 | 119.716 | S(16)-C(12)-C(8) | 116.011 | 116.779 |
| C(23)-C(22)-C(21) | 120.231 | 120.008 | N(13)-C(12)-C(8) | 125.168 | 124.958 |
| C(22)-C(21)-C(14) | 120.776 | 120.987 | N(20)-C(11)-S(17) | 74.448 | 71.333 |
| C(30)-N(20)-C(19) | 118.816 | 118.687 | N(20)-C(11)-C(10) | 123.65 | 123.532 |
| C(30)-N(20)-C(11) | 119.085 | 119.021 | S(17)-C(11)-C(10) | 87.099 | 84.163 |
| C(19)-N(20)-C(11) | 122.091 | 122.056 | C(11)-C(10)-C(9) | 123.236 | 123.633 |
| C(28)-C(19)-N(20) | 119.066 | 119.244 | C(10)-C(9)-C(1) | 126.397 | 125.604 |
| C(28)-C(19)-C(18) | 120.267 | 120.125 | C(12)-C(8)-C(7) | 123.797 | 122.951 |
| N(20)-C(19)-C(18) | 120.472 | 120.599 | C(8)-C(7)-C(4) | 125.561 | 126.251 |
| C(25)-C(18)-C(19) | 117.879 | 117.988 | C(5)-C(6)-C(1) | 121.626 | 121.685 |
| C(25)-C(18)-S(17) | 119.433 | 119.2 | C(6)-C(5)-C(4) | 121.531 | 121.48 |
| C(19)-C(18)-S(17) | 122.536 | 122.68 | C(7)-C(4)-C(5) | 123.084 | 123.428 |
| C(18)-S(17)-C(11) | 71.064 | 69.516 | C(7)-C(4)-C(3) | 120.061 | 119.766 |
| C(15)-S(16)-C(12) | 71.167 | 70.169 | C(5)-C(4)-C(3) | 116.839 | 116.795 |
| C(24)-C(15)-S(16) | 118.172 | 118.121 | C(4)-C(3)-C(2) | 121.612 | 121.719 |
| C(24)-C(15)-C(14) | 118.074 | 118.105 | C(3)-C(2)-C(1) | 121.415 | 121.399 |
| S(16)-C(15)-C(14) | 123.682 | 123.458 | C(9)-C(1)-C(6) | 123.263 | 122.958 |
| C(21)-C(14)-C(15) | 119.577 | 119.506 | C(9)-C(1)-C(2) | 119.847 | 120.2 |
| C(21)-C(14)-N(13) | 119.013 | 119.36 | C(6)-C(1)-C(2) | 116.886 | 116.842 |
